# ZnO Additive Boosts Charging Speed and Cycling Stability of Electrolytic Zn–Mn Batteries

**DOI:** 10.1007/s40820-023-01296-y

**Published:** 2024-01-04

**Authors:** Jin Wu, Yang Tang, Haohang Xu, Guandie Ma, Jinhong Jiang, Changpeng Xian, Maowen Xu, Shu-Juan Bao, Hao Chen

**Affiliations:** Institute for Clean Energy & Advanced Materials, School of Materials and Energy, Southwest University, Chongqing, 400715 People’s Republic of China

**Keywords:** Electrolytic aqueous zinc-manganese batteries, Electrolyte pH value, ZnO electrolyte additive, Fast constant-voltage charging ability

## Abstract

Electrolytic aqueous zinc-manganese (Zn–Mn) batteries have the advantage of high discharge voltage and high capacity due to two-electron reactions. However, the pitfall of electrolytic Zn–Mn batteries is the sluggish deposition reaction kinetics of manganese oxide during the charge process and short cycle life. We show that, incorporating ZnO electrolyte additive can form a neutral and highly viscous gel-like electrolyte and render a new form of electrolytic Zn–Mn batteries with significantly improved charging capabilities. Specifically, the ZnO gel-like electrolyte activates the zinc sulfate hydroxide hydrate assisted Mn^2+^ deposition reaction and induces phase and structure change of the deposited manganese oxide (Zn_2_Mn_3_O_8_**·**H_2_O nanorods array), resulting in a significant enhancement of the charge capability and discharge efficiency. The charge capacity increases to 2.5 mAh cm^−2^ after 1 h constant-voltage charging at 2.0 V *vs.* Zn/Zn^2+^, and the capacity can retain for up to 2000 cycles with negligible attenuation. This research lays the foundation for the advancement of electrolytic Zn–Mn batteries with enhanced charging capability.

**Supplementary Information:**

The online version contains supplementary material available at 10.1007/s40820-023-01296-y.

## Highlights

- Low pH value of electrolyte suppresses the charge capabilities of electrolytic Zn–Mn batteries.
- Unique solid phase alkaline properties of zinc sulfate hydroxide hydrate endow the electrolytic Zn–Mn batteries with greatly enhanced charge capabilities.
- The highly active Zn_2_Mn_3_O_8_·H_2_O nanorods array deposited during the charge process improve the discharge efficiency and stability of electrolytic Zn–Mn batteries.

**Supplementary Information:**

The online version contains supplementary material available at 10.1007/s40820-023-01296-y.

## Graphical abstract

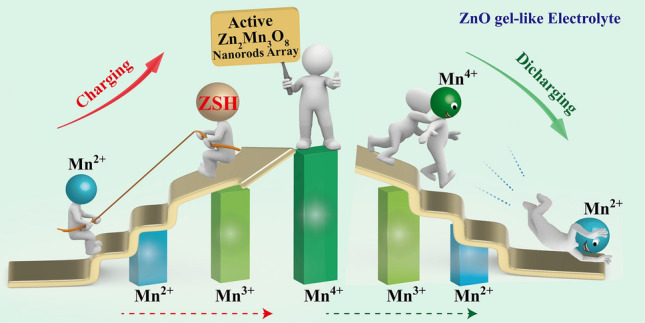

## Introduction

**Fig. 1:**
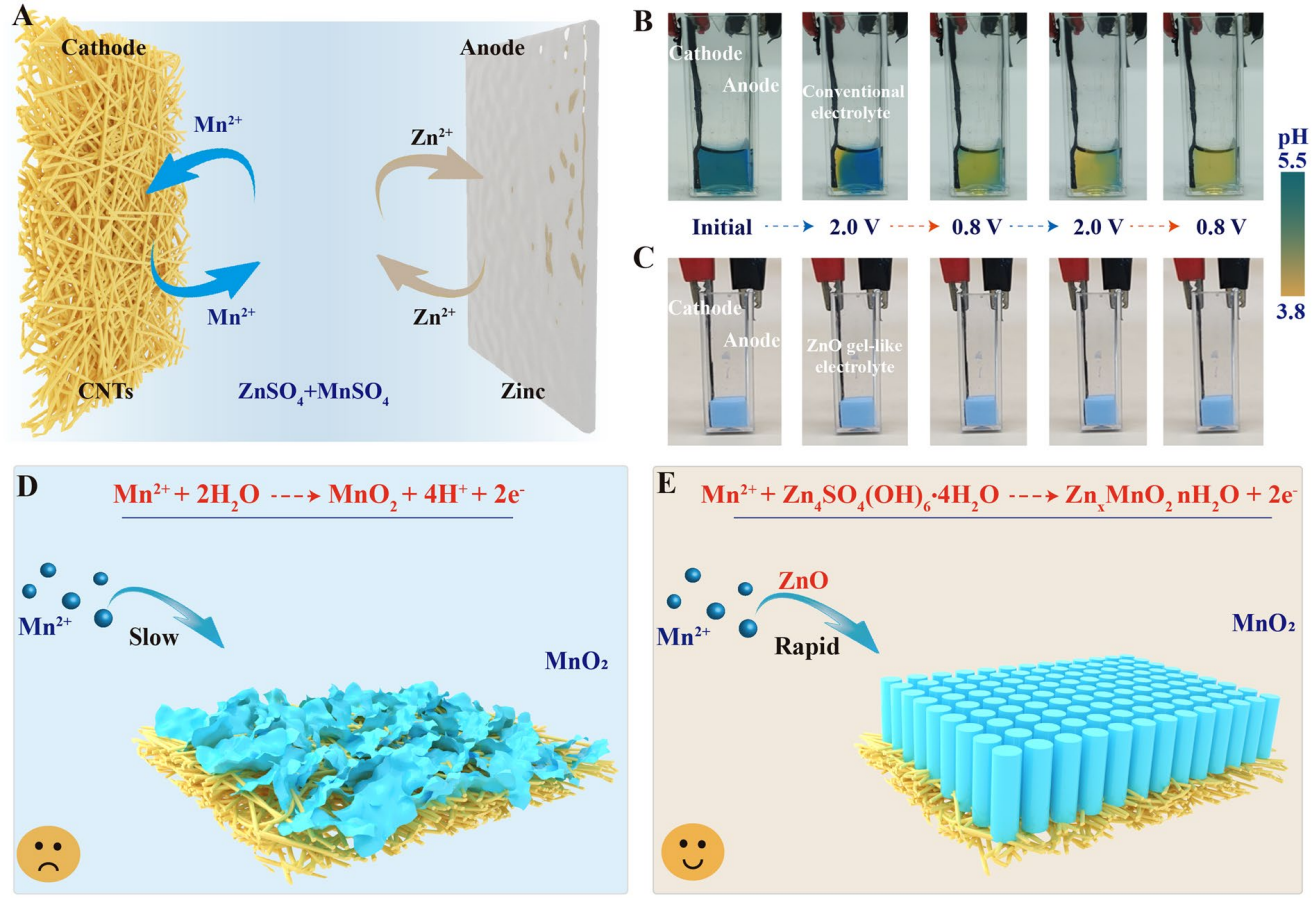
Reaction process and problem analysis of electrolytic Zn–Mn batteries. **A** Schematic illustrating the electrolytic Zn–Mn battery components and the electrolytic charging and discharging reactions. **B**, **C** Digital images depicting the transparent Zn–Mn cell with ZnSO_4_ + MnSO_4_ auqoues electrolyte and ZnO gel-like electrolyte at various stages of full charge/discharge, with the black clamp on the right corresponding to the negative zinc electrode and the red clamp on the left corresponding to the positive CNT film-based electrode. Bromocresol green, a pH indicator with sensitivity in the pH range of 3.8–5.5 was added to the electrolyte solution. Schematics depicting cathode deposition reaction states in **D** conventional ZnSO_4_ + MnSO_4_ aqueous electrolyte, and **E** ZnO gel-like electrolyte

Rechargeable aqueous zinc-manganese (Zn–Mn) batteries have emerged as a research hotspot in the field of grid-scale energy storage systems (EESs) due to exceptional safety feature, economical nature and nontoxicity [1–12]. Among them, electrolytic Zn–Mn battery based on deposition-dissolution reactions receives increasing attentions as it delivers higher capacity due to two-electron redox reactions than conventional Zn–MnO_2_ batteries system based on one-electron reaction [13–20]. During the electrolytic Zn–Mn battery charging process, solid manganese oxide form through electrochemical deposition reaction, which then dissolves during discharging (Fig. 1A) [21–25]. An electrolytic Zn–Mn battery incorporating an acidic electrolyte containing ZnSO_4_ + MnSO_4_ and an H_2_SO_4_ additive exhibits high cathode reaction reversibility and a long cycle life (2000 cycles) [26]. Subsequently, in order to mitigate corrosion of the zinc mental anode in the strong acidic electrolyte and broaden the electrochemical window, researchers explored decoupled batteries by using ion-selective exchange membranes to separate cathode and anode electrolytes [27–29]. This kind of electrolytic Zn–Mn battery represents a technological step forward to realize low-cost and high-safety stationary energy storage [30–32].

Previous research has made remarkable progress on improving the discharge performance of electrolytic Zn–Mn batteries using acidic electrolytes [33, 34]. However, few studies have been performed to chronoamperometric charge performances. Here, we found that with increasing electrolyte acidity, more limited Mn^2+^ deposition was observed during the charging process. As illustrated in Fig. S1, after 30 min chronoamperometric charging at 2.0 V *vs.* Zn/Zn^2+^, the acidic electrolytic Zn–Mn batteries exhibit low charge capacities (0.14 mAh cm^−2^ and 0.17 mAh cm^−2^ with electrolytes pH values of 1 and 2, respectively). This indicates that acidic electrolytes are unfavorable for manganese oxides deposition reactions, as indicated in the equation [35–37]:

$${{\text{Mn}}}^{2+}+{2{\text{H}}}_{2}{\text{O}}\to {{\text{MnO}}}_{2}+{4{\text{H}}}^{+}+{2{\text{e}}}^{-}$$

Furthermore, the pH of a mild-acidic ZnSO_4_ + MnSO_4_ electrolyte during the charge–discharge process is unstable. This has been confirmed by using a transparent electrolytic Zn–Mn cell (Fig. 1B). The initial color of the electrolyte is light blue (pH value of 4.6), which changes to yellow after first 30 min chronoamperometric charging process. With continued cycling, the entire electrolyte turned light yellow (pH value of 3.0). This result indicates that the manganese oxide electrodeposition process generates H^+^, which ultimately will slow down the subsequent manganese oxide electrodeposition. Hence, the conventional ZnSO_4_ + MnSO_4_ electrolytes cannot guarantee fast manganese oxide deposition reaction kinetics, and maintaining a stable and suitable electrolyte chemical environment may benefit the charge capability for electrolytic Zn–Mn batteries (shown in Fig. 1D).

We previously shown that the high OH^−^ content of layered Zn_4_SO_4_(OH)_6_·nH_2_O (ZSH) enables itself to act as a highly basic electrolyte to induce rapid Mn^2+^ deposition from an initial voltage of 1.5 V *vs.* Zn/Zn^2+^ (Eq. S1) [38]. Herein, we develop a unique gel-like electrolyte by incorporating ZnO powder into the solution of 1 M ZnSO_4_ + 2 M MnSO_4_, in which the ZSH forms spontaneously within the electrolyte (Eqs. S4–S6).The transparent electrolytic Zn–Mn cell shown that the ZnO gel-like ekectrolyte exhibit stable pH value (6.4) during the cycling (Fig. 1C). The ZnO gel-like electrolyte is applied for the fabrication of an electrolytic Zn–Mn battery with a carbon nanotube film (CNTs) cathode substrate and a zinc foil anode (Fig. 1E). This battery only requires 0.6 h to achieve a charge capacity of 2.5 mAh cm^−2^ at a chronoamperometric charge voltage of 2.0 V *vs.* Zn/Zn^2+^, and the time decreases to 2.5 h after 100 cycles. In contrast, a battery incorporating the conventional liquid aqueous solution of 1 M ZnSO_4_ + 2 M MnSO_4_ requires 12.5 h to achieve a charge capacity of 2.5 mAh cm^−2^ after 100 cycles. The manganese oxide deposition reaction and associated structure and phase transformation will be presented in details for the understanding of the charge storage mechanism, which differs significantly from conventional Zn–Mn electrolytic battery.

## Results and Discussion

### Preparation and Properties of the Gel Electrolyte

**Fig. 2:**
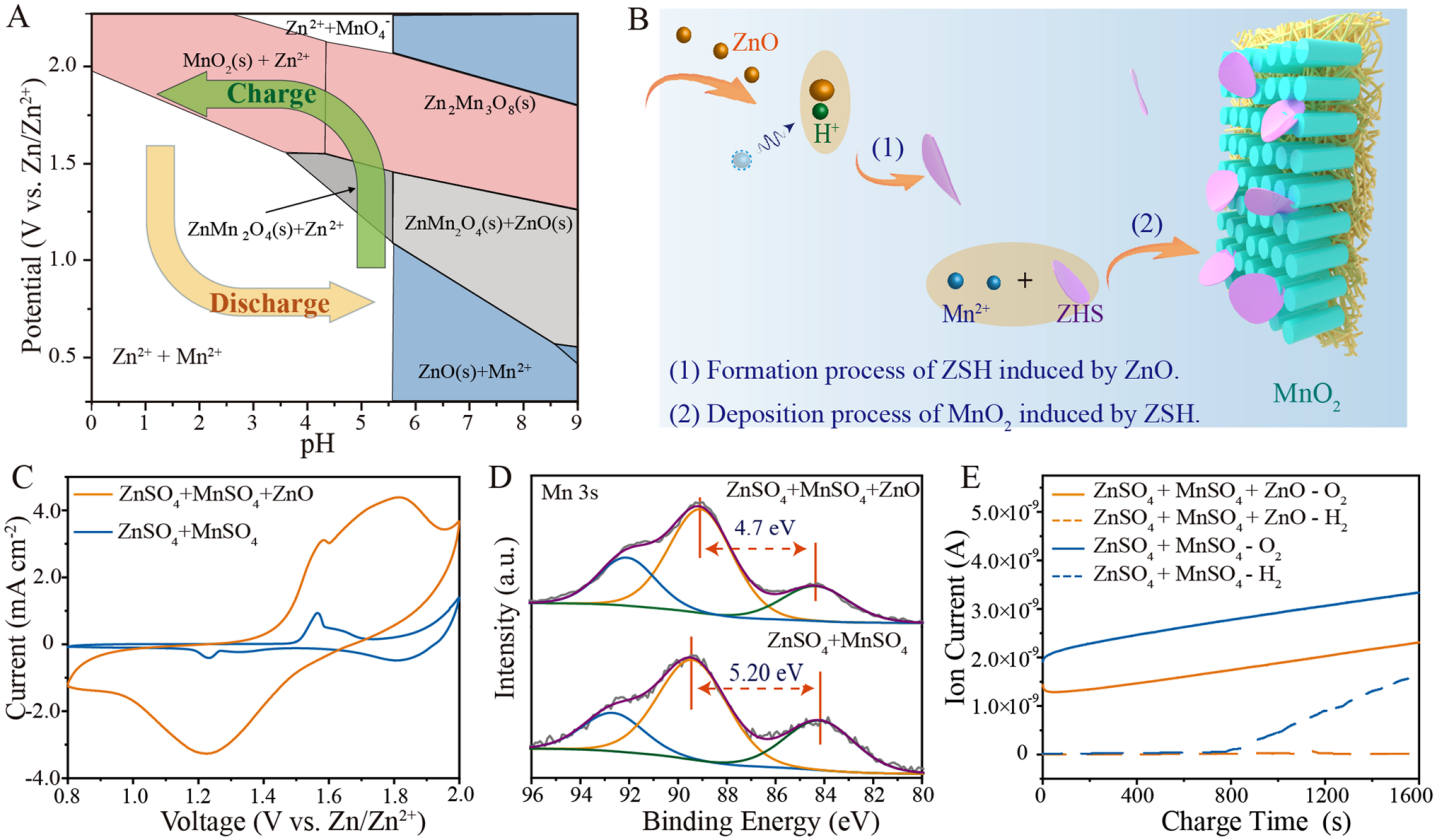
Analysis of reasons for charging capability enhancement. **A** Pourbaix diagrams depicting zinc and manganese chemical reactions in a 1 M Zn^2+^  + 2 M Mn^2+^ electrolyte. **B** Schematic illustrating the mechanism underlying the promotion of Mn^2+^ electrodeposition by a ZnO gel-like electrolyte. **C** Cyclic voltammetry (CV) curves generated using a scanning rate of 1 mV s^−1^ for electrolytic Zn–Mn coin cell batteries with and without the ZnO electrolyte additive. **D** High-resolution Mn 3*s* XPS spectra of CNTs cathode in gel-like electrolyte (top) or aqueous electrolyte (bottom) after first chronoamperometric charge process (2 V *vs.* Zn/Zn^2+^ for 30 min). **E** In-situ differential electrochemical mass spectrometry (DEMS) gas analysis curves obtained from different electrolytic Zn–Mn batteries during the chronoamperometric charge process (2 V *vs.* Zn/Zn^2+^). The blue line represents H_2_ and the orange line represents O_2_

According to the Pourbaix diagram depicting zinc and manganese chemical reactions (shown in Fig. 2A; Get from an Open-Source calculation system of THE MATERIALS PROJECT), pH increase under the same charging potential promotes the formation of higher-valence manganese oxide. For the conventional ZnSO_4_ + MnSO_4_ electrolyte, the significant drop in pH at the cathode surface during the charging process (green arrow) leads to reduced Mn^2+^ deposition ability. Therefore, maintaining a high electrolyte pH environment can significantly improve Zn–Mn battery charge performance. Previously reported results indicated that ZSH is a highly alkaline unstable layered hydrate that can accelerate electrodeposition of zinc-containing manganese oxides (Eq. S1 and Fig. 2B). Thus, using ZSH as an electrolyte additive has great potential for enhancing battery charging capability.

A commercial film consisting of CNTs was selected as the cathode substrate for this study. Figure S2 depicts the network of interconnected carbon nanotubes comprising the highly flexible film, which contains 0.57% metal iron catalyst by weight. A ZnO gel-like electrolyte was prepared by mixing ZnO nanoparticles with a solution consisting of 1 M ZnSO_4_ + 2 M MnSO_4_. Figure S3 illustrates the physical transformation of the mixture from a fluid state to a dense, white, gelatinous state, with the ionic conductivity of 22.693 mS cm^−2^ (Table S1). After washing and drying, the insoluble substance of the ZnO gel-like electrolyte shows numerous flakes measuring several microns in length (Fig. S4A, B). The X-ray diffraction (XRD) pattern confirmed the existence of two new phases, Zn_4_SO_4_(OH)_6_·4H_2_O and Zn_4_SO_4_(OH)_6_ 3H_2_O (ZSH; PDF # 44–0673 and 39-0689; shown in the Fig. S5), thus indicating that ZnO nanoparticles were transformed into ZSH in the 1 M ZnSO_4_ + 2 M MnSO_4_ aqueous solution.

To verify the electrochemical performance of ZnO gel-like electrolyte, different batteries incorporating the cathode CNT film substrate and Zn foil as anode were assembled with either liquid aqueous electrolyte (1 M ZnSO_4_ + 2 M MnSO_4_) or with the ZnO gel-like electrolyte (1 M ZnSO_4_ + 2 M MnSO_4_ + ZnO). Thereafter, a CR2032 coin-type cell mold was utilized to construct the batteries. In Fig. 2C, the CV curve obtained for the battery without the ZnO additive exhibits a pair of redox peaks at 1.56 and 1.23 V *vs.* Zn/Zn^2+^. By contrast, the battery incorporating the ZnO electrolyte yields significantly higher current and a broader oxidation peak at a higher potential, thus demonstrating enhanced capacity. After 90-min chronoamperometric charge of 2 V *vs.* Zn/Zn^2+^, the CNT cathode film was removed, cleaned, and subjected to XPS analysis (Fig. 2D). Energy separation (ΔE) value between the two Mn 3*s* peaks is 5.2 eV for the cathode with conventional aqueous electrolyte and 4.7 eV for the cathode with ZnO gel-like electrolyte. Based on the linear relationship between Mn chemical valence and Mn 3*s* ΔE value [39, 40], the average oxidation state of Mn is around 3.34 and 4 for cathodes without and with ZnO additive, respectively. This indicates that the manganese oxide deposited from the ZnO gel-like electrolyte exhibits a higher valence than that deposited in the cathode with conventional aqueous electrolyte. The results of in-situ differential electrochemical mass spectrometry (DEMS) analysis of gases produced during the chronoamperometric charge process (Fig. 2E) shows that the battery with the ZnO gel-like electrolyte release less O_2_ from the cathode and H_2_ from the anode during the First charging process. The the linear sweep voltammetry curves (Fig. S6) further comfirm this. A soft packed battery was assembled to evaluate the gas evolution reaction during cycling (Fig. S7). By incorporating ZnO additives, the gas production of the battery significantly reduces from 14.7 to 0.91 cm ^−3^ after 100 cycles. The reduction in gas release suggests improved battery safety during practical applications. Taken together, the abovementioned results suggest that the ZnO gel-like electrolyte can effectively enhance Mn^2+^ electrodeposition and reduce gas generation.

### Morphology Evolution and Phase Transformation

**Fig. 3:**
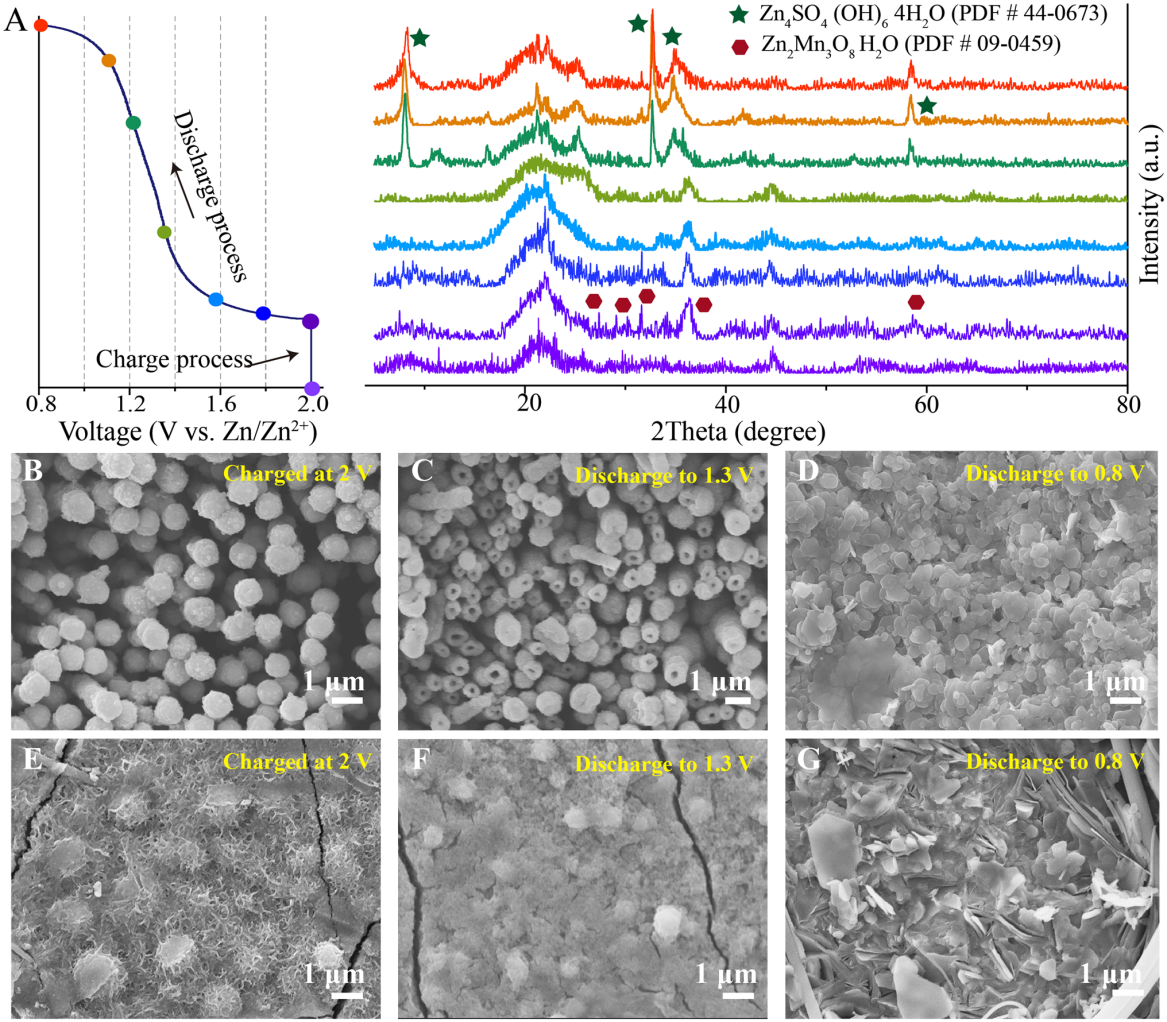
Phase and structure evolution of the cathode. **A** Ex-situ XRD pattern of cathode surface during the charge–discharge process in the electrolytic Zn–Mn battery containing the ZnO gel-like electrolyte (2.0 V *vs.* Zn/Zn^2+^ chronoamperometric charge capacity of 1.5 mAh cm^−2^, discharge current of 0.1 mA cm^−2^). The corresponding FESEM images of cathode surface at different charge and discharge states (30 min chronoamperometric charge at 2.0 V *vs.* Zn/Zn^2+^, discharge current of 0.1 mA cm^−2^): **B**–**D** ZnO gel-like electrolyte and **E**–**G** conventional aqueous electrolyte

To clearly demonstrate the phase evolution process occurring on cathode surface, an electrolytic Zn–Mn battery with ZnO gel-like electrolyte was charged to 1.5 mAh cm^−2^ using a chronoamperometric charge of 2 V *vs.* Zn/Zn^2+^ then discharged to 0.8 V *vs.* Zn/Zn^2+^ at 0.1 mA cm^−2^ followed by collection of ex situ XRD patterns (shown in Fig. 3A). Except the diffraction of CNTs (around 23° and 45°), weak reflection peaks of Zn_2_Mn_3_O_8_ (PDF#09-0459) appears upon completion of the charging process (Fig. S8). Furthermore, characteristic ZSH peaks (PDF#44-0673) gradually appear and characteristic Zn_2_Mn_3_O_8_ peaks gradually disappear as the battery is discharged to 0.8 V *vs.* Zn/Zn^2+^, as consistent with results of our previous studies that periodic ZSH emergence participates in the charge storage process [38]. In the conventional aqueous electrolyte, the deposited manganese oxides on the CNTs have low crystallinity and no obvious characteristic XRD diffraction peak can be observed after a chronoamperometric charge of 2.0 V *vs.* Zn/Zn^2+^ (Fig. S9).

The morphological evolution at the cathode surface during the charge/discharge process was investigated by applying a 30 min chronoamperometric charge of 2 V *vs.* Zn/Zn^2+^ followed by discharge to 0.8 V at 0.1 mA cm^−2^, and the results were summarized in the Fig. S11. After the charging process, the cathode surface of the battery containing the ZnO gel-like electrolyte is uniformly covered with a nanorod array (Figs. 3B and S12), and the length of nanorods increases with the increasing deposition capacity (Fig. S13). However, a sharp different morphology is observed for the battery containing the conventional aqueous electrolyte, with uniform and slack nanosheets deposited on the cathode surface (Fig. 3E). Moreover, the mass of manganese oxide deposited in the ZnO gel-like electrolyte has around threefold greater than deposited in the convention aqueous electrolyte (Table S2). After batteries are discharged to 1.3 V *vs.* Zn/Zn^2+^, the array remains and manganese oxide nanorods gradually become hollow (Fig. 3C). Further discharge to 0.8 V *vs.* Zn/Zn^2+^ leads to dissolution of all hollow nanorods followed by their transformation into homogeneous small ZSH flakes (Fig. 3D). The more details and discutions about disolution process of deposited manganese oxide were presented in the Figs. S14 and S15. In contrast, the cathode surface of the battery containing conventional aqueous electrolyte undergoes a different transformation, where the deposited manganese oxide nanosheets nearly disappear to expose numerous particles after discharge to 1.3 V (Fig. 3F). Subsequent discharge to 0.8 V results in the emergence of staggered flakes (Fig. 3G).

**Fig. 4:**
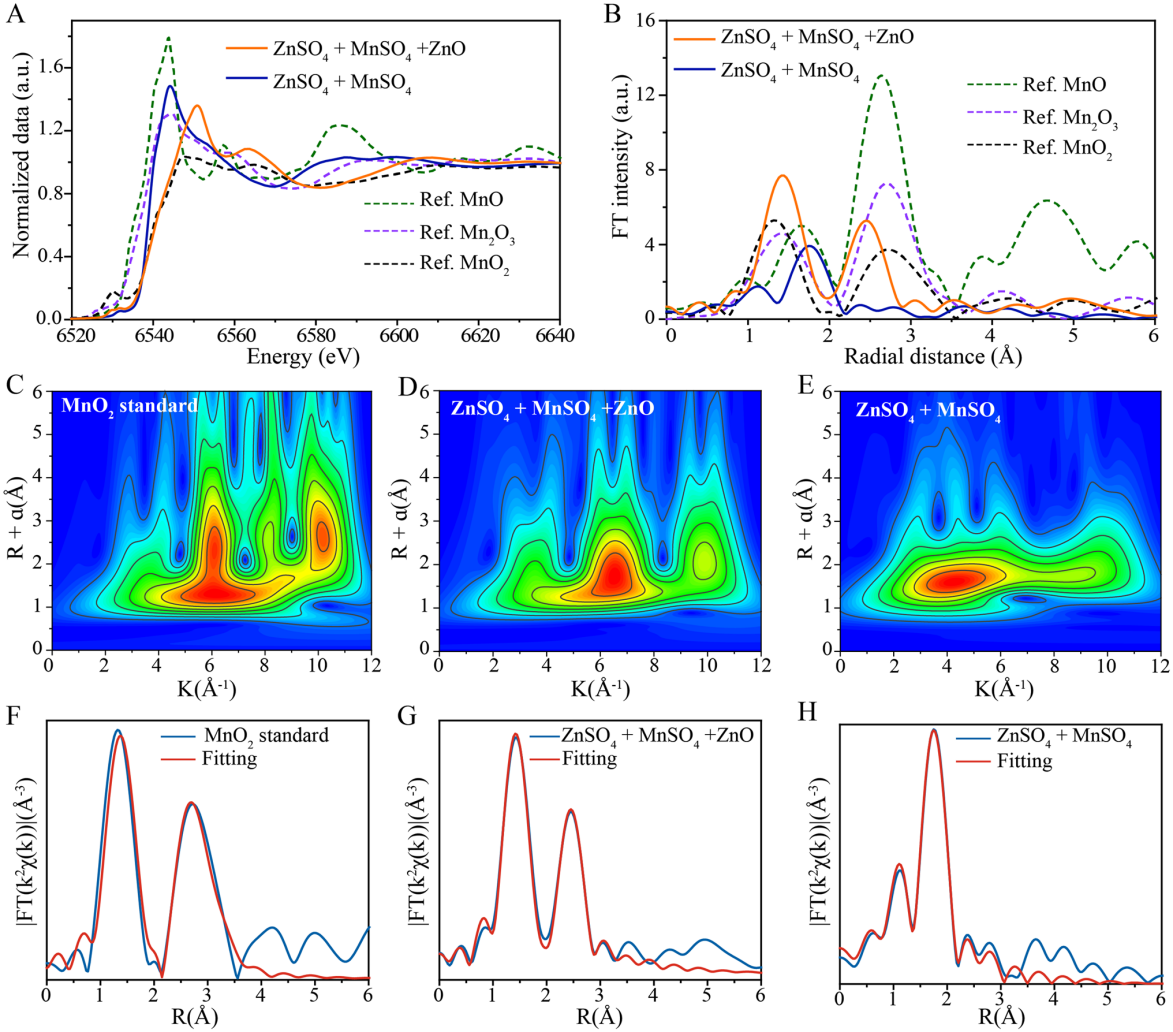
Mn K-edge X-ray absorption fine structure (XAFS) analysis of the deposited manganese oxide. **A** Normalized Mn K-edge XANES spectra and **B** EXAFS spectra in R-spaces of the MnO standard, Mn_2_O_3_ standard, MnO_2_ standard, full-charge state cathode CNTs in different electrolyte. WT for k^3^-weighted EXAFS signals of **C** the MnO_2_ standard with the CNTs cathode, **D** the ZnO gel-like electrolyte, and **E** aqueous electrolyte at the Mn K-edge. Fourier transform of the k^3^-weighted EXAFS spectrum and fit in R-space of the **F** MnO_2_ standard and the CNTs cathode immersed in **G** gel-like electrolyte and **H** aqueous electrolyte at the Mn K-edge; the CNTs cathode received a chronoamperometric charge of 2.0 V *vs.* Zn/Zn^2+^ for 30 min

X-ray absorption fine structure (XAFS) analysis of the Mn K-edge was conducted to determine the electronic structure of deposited manganese oxides in different electrolytes, with normalized Mn K-edge X-ray absorption near edge structure (XANES) spectra shown in Fig. 4A. In the No gel-like electrolyte, the XANES spectrum of deposited Mn on cathode CNTs after charging displays a shift in the position of absorption edge towards higher energy as compared to that of the battery containing conventional aqueous electrolyte. This result indicates that the valence state of Mn deposited in the Zano gel-like electrolyte was higher than that deposited in the conventional aqueous electrolyte alone. Furthermore, comparisons of these results to those obtained for standard manganese (II/III/IV) oxides revealed that the XANES spectrum of manganese oxide deposited from the conventional aqueous electrolyte was similar to that obtained for the standard Mn_2_O_3_ sample, while the XANES spectrum of manganese oxide deposited from the Zano gel-like electrolyte resembled that obtained for the standard MnO_2_ sample [41–45].

To gain a deeper understanding of the local structure surrounding Mn centers, extended XAFS (EXAFS) spectra are Fourier transformed into the radial space (R-space) in order to allow for discriminatory changes within each coordination sphere. Figure 4B shows EXAFS spectra of the aforementioned samples in R-space, with the first major peak observed at approximately 1.5 Å, which corresponds to the first-shell Mn–O scattering path within the solid MnO_2_ structure. The second major peak observed at around 2.5 Å corresponds to the second-shell Mn–Mn scattering path. Notably, the peaks of charging products in ZnO gel-like electrolyte were similar to those of Mn_2_O_3_, with the peak at approximately 1.5 Å corresponding to the first-shell Mn–O scattering path. Conversely, charge products in the aqueous electrolyte compared to MnO, with the peak at approximately 1.7 Å corresponding to the first-shell Mn–O scattering path showing a decrease in FT peak amplitude and position and a change towards a longer bond distance. Hence, there exists a significant difference in MnO_2_ coordination number between the two electrolyte types [46–48].

Wavelet transform (WT) analysis was next conducted to study Mn K-edge EXAFS oscillations. WT contour plots with intensity maximums located at coordinates of (k, R), which are closely related to the path length R and atomic number Z, can provide significant clues that distinguish the coordination structures. Figure 4C shows an intensity maximum at (6.1 Å^−1^, 1.5 Å) for the MnO_2_ standard, which is smaller in both wave vector k and bond length R as compared to that of Mn atoms of manganese oxides deposited within the ZnO gel-like electrolyte (6.5 Å^−1^, 1.6 Å), as shown in Fig. 4D. This result indicates a lower coordinated atomic number and shorter bond length for Mn atoms in manganese oxides deposited within the ZnO gel-like electrolyte. By contrast, as show in the Fig. 4E, the bond length R relative to Mn in manganese oxides deposited within the ZnO gel-like electrolyte (6.5 Å^−1^, 1.6 Å) is similar to that of manganese oxides deposited within the aqueous electrolyte (4.2 Å^−1^, 1.6 Å), an effect that may be attributed to the presence of the Zn atom.

We next fit the main FT peaks observed from 1 to 3 Å (Fig. 4F–H) then utilized EXAFS structural parameters (see Table S3) and several theoretical structures to perform calculations to determine molecular structures of deposits using the FEFF program. Theoretical structures included those of MnO (cif # mp-19006), MnO_2_ (cif # mp-19395), ZnMn_2_O_4_ (cif # mp-18751), and Zn_2_Mn_3_O_8_ (cif # mp-1042798) (Fig. S18A-D). As presented in Table S3, the molecular form of manganese oxide deposited during charging in the ZnO gel-like electrolyte was consistent with the model Zn_2_Mn_3_O_8_ (cif # mp-1042798), as based on the atomic distance between Zn–Mn atoms. However, the phase of manganese oxide deposited within the aqueous electrolyte was difficult to determine, due to its poor crystallinity, complex composition, and the long Zn–Mn atomic distance. Nevertheless, we conclude that most of the deposited material mass in the aqueous electrolyte consisted of ZnMn_2_O_4_ (cif # mp-18751), with a smaller mass of deposited MnO (cif # mp-19006) present in the mixture.

To conclude, after the comprehensive analysis, we suggest that the manganese oxide deposited in the ZnO gel-like electrolyte predominantly consists of Zn_2_Mn_3_O_8_ H_2_O. The deposits are in the form of a uniform nanorod array structure and the deposition mass is around 3 times greater than that of manganese oxide nanosheets deposited within the conventional aqueous electrolyte (ZnMn_2_O_4_). Moreover, during the discharge process, hollowing of the Zn_2_Mn_3_O_8_ H_2_O nanorod occurs, which can enhance the ion penetration and diffusion and to ultimately lead to superior discharge efficiency and stability.

### Performance of Electrolytic Zn–Mn Battery

**Fig. 5:**
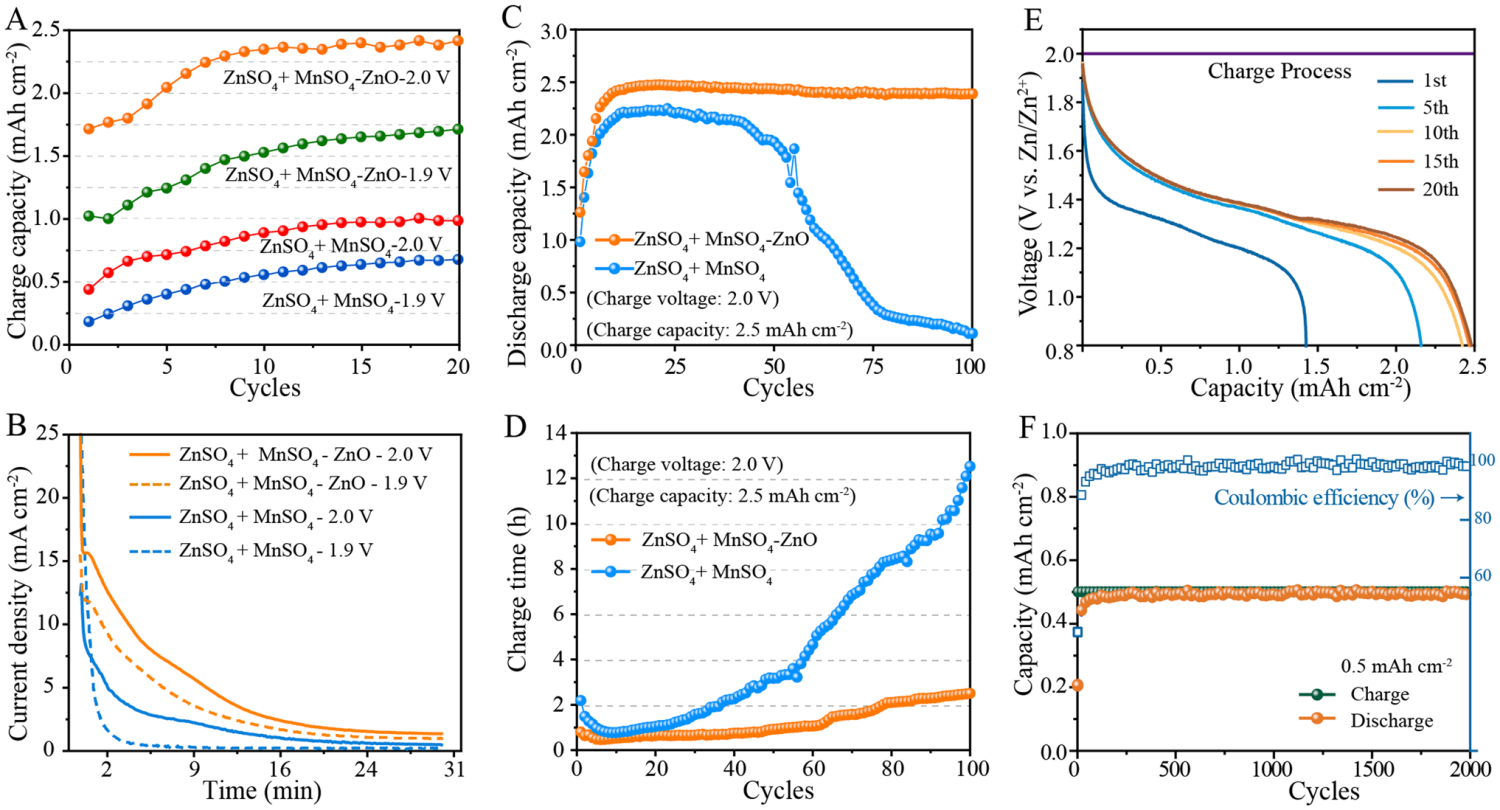
Electrochemical performances of different electrolytic Zn–Mn button batteries. **A** Charge capacities of different batteries during the initial 20 cycles at a chronoamperometric charge under 2.0 V *vs.* Zn/Zn^2+^ for 30 min. **B** Charge current variations of different batteries under different voltages for 30 min during the first charge process. **C** Discharge capacity and **D** corresponding charge time during 100 cycles (charge under 2.0 V *vs.* Zn/Zn^2+^ to 2.5 mAh cm^−2^ and discharge at 0.5 mA cm^−2^ at 0.8 V *vs.* Zn/Zn^2+^). **E** Galvanostatic discharge curves of the ZnO gel-like electrolyte battery (charge under 2.0 V *vs.* Zn/Zn^2+^ to 2.5 mAh cm^−2^ and discharge at 0.5 mA cm^−2^). **F** Long-term cycling performance (charge of 2.0 V *vs.* Zn/Zn^2+^ to 0.5 mAh cm^−2^ and discharge at 1 mA cm^−2^ to 0.8 V *vs.* Zn/Zn^2+^)

The detail electrochemical performances of electrolytic Zn–Mn battery incorporating the ZnO gel-like electrolyte were collected. In Fig. 5A, it is evident that after 20 cycles, the charge capacity of the ZnO gel-like electrolyte-containing Zn–Mn battery significantly increases and reaches 1.73 and 2.48 mAh cm^−2^ after 30 min chronoamperometric charging at 1.9 and 2.0 V *vs.* Zn/Zn^2+^, respectively, as compared to charge capacities of only 0.62 and 1.0 mAh cm^−2^, respectively, for the batteries containing conventional aqueous electrolyte. We also recorded the working current during the first chronoamperometric charge process and observed substantially increased charge current for the gel-electrolyte battery as compared to the conventional aqueous electrolyte battery. Additionally, we note that the late-stage charge current remains stable above 1.5 mA cm^−2^ in the former battery, while the latter is approximately 0 (Fig. 5B). These results suggest that Mn^2+^ deposition occurs continuously in the gel-like electrolyte but only occurs during the initial charge stage in the conventional aqueous electrolyte.

We conducted testing of battery cycling performance at high specific surface capacity and low current density discharge conditions. The test method involved chronoamperometric charging to 2.5 mAh cm^−2^ at 2.0 V *vs.* Zn/Zn^2+^ followed by 0.5 mA cm^−2^ constant current discharge to 0.8 V *vs.* Zn/Zn^2+^. As shown in Fig. 5C, E, the discharge specific capacity continues to increase during the first 10 cycles and reaches 2.48 and 2.3 mAh cm^−2^ in batteries containing gel-like electrolyte and conventional aqueous electrolyte, respectively. After 100 cycles, the discharge capacity remains at 2.40 mAh cm^−2^ in the gel-like electrolyte, while the discharge capacity is only 0.11 mAh cm^−2^ in the conventional aqueous one. The chronoamperometric charge time was collected during the cycling (shown in Fig. 5D). It is evident that the chronoamperometric charge time gradually decreases to 0.6 h from 1 h after several cycles and then increases to 2.5 h after 100 cycles for the gel-electrolyte batteries which is shorter than that observed for conventional aqueous electrolyte batteries (from 2.3 to 12.5 h). We further studied long-cycle stability. As shown in Fig. 5F, the gel-electrolyte batteries demonstrate outstanding stability without noticeable capacity decay even after 2000 cycles. Obviously, the high discharge stability correlates with excellent chronoamperometric charge ability. Furthermore, the superior stability of zinc metal anode also effectively prolongs the cycle life of the electrolytic Zn–Mn batteries with ZnO gel-like electrlyte (Fig. S24).

**Fig. 6:**
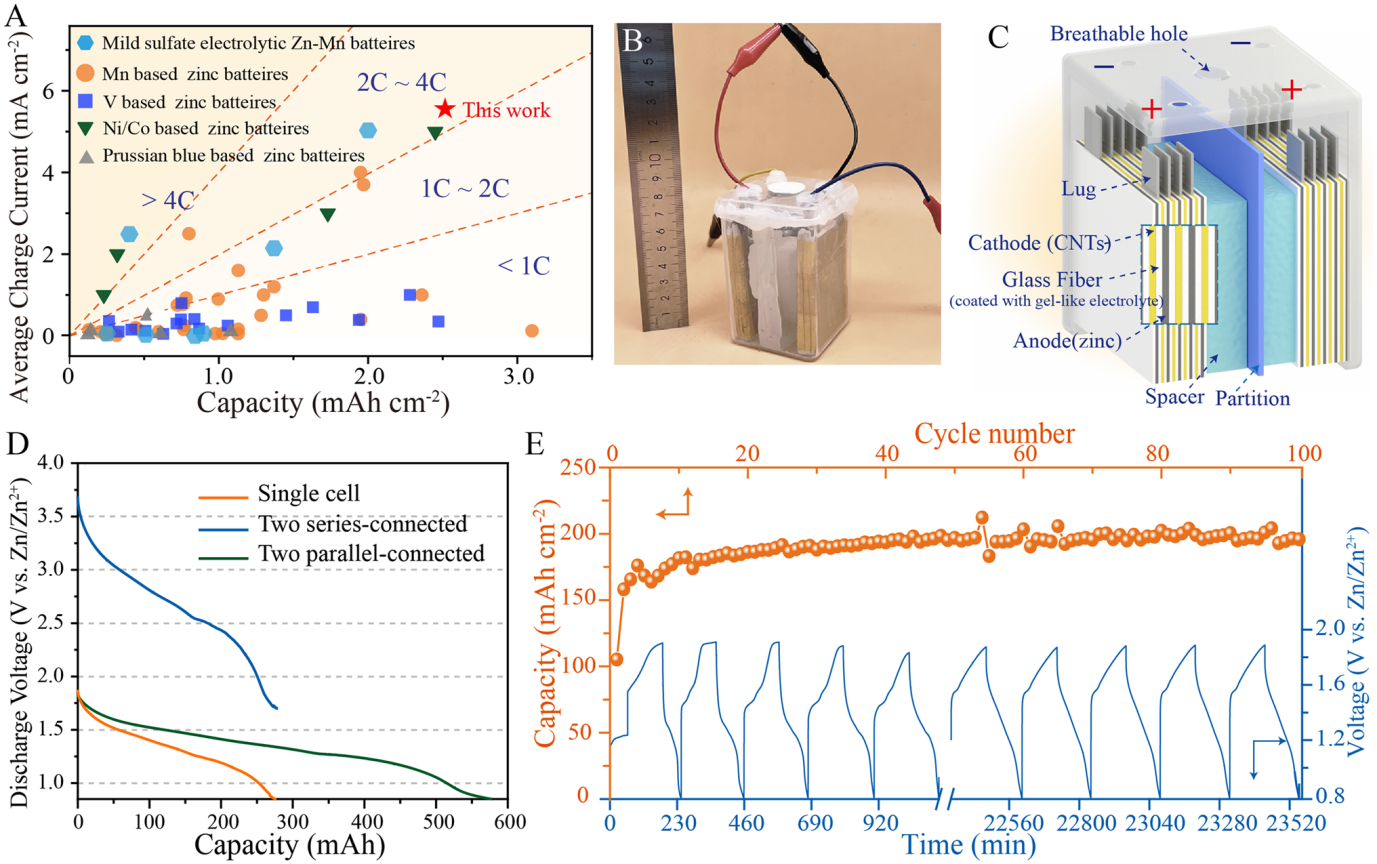
Performance of square shell electrolytic Zn–Mn cell. **A** Areal specific capacities and corresponding charge current values of state-of-the-art Zn aqueous battery systems (calculations based on cathode active material mass). **B** Digital image of electrolytic Zn–Mn square shell cell. **C** Schematic diagram of electrolytic Zn–Mn square shell cell. **D** Discharge curves of decoupled electrolytic Zn–Mn square shell cells containing a ZnO gel-like electrolyte, including single cell, two cells in series, and two cells in parallel. The discharge current is 50 mA for both the single cell and for two cells in series, and 100 mA for two cells in parallel. **E** Cycling stability test of assembled square shell cells (charge to constant capacity of 200 mAh, discharge current of 100 mA)

A comprehensive comparison of charge areal specific capacities and corresponding average charge currents of representative zinc-based aqueous batteries and electrolytic Zn–Mn batteires were presented in Fig. 6A (further details are provided in Tables S4 and S5). Different color saturation levels correspond to different charging speeds, with higher capacities correlating with greater electrochemical performance at a given charge speed. Notably, as compared to other reported zinc-based batteries systems, our electrolytic Zn–Mn batteries provide significant advantages in terms of both areal specific capacity and charge rate capability by achieving a charge capacity of 2.5 mAh cm^−2^ and a maximum charge rate of 2C.

Toward achieving industrial application, we developed an electrolytic Zn–Mn battery prototype with a 600 mAh charge capacity that is encased in a square shell package (8 cm × 20 cm × 2 cm). The battery cathode was assembled from four CNT slices and the anode from five Zn metal slices, with anode and cathode separated by glass fiber coated with a ZnO gel-like electrolyte (Fig. 6B, C). When two cells are connected in series, the output voltage is doubled without sacrificing capacity (Fig. 6D). Alternatively, when two cells are connected in parallel, the battery exhibits a doubled discharge capacity of 585 mAh. We also evaluated long-term cycling performance of a single square shell cell (Fig. 6E). During initial cycles, the discharge capacity continually increases then peaks at 20 cycles and remains stable thereafter.

## Conclusion

We have shown that an aqueous electrolytic Zn–Mn battery incorporating a ZnO gel-like electrolyte exhibits significantly enhanced chronoamperometric charge capability and cycling stability compared to conventional sulfite electrolytic Zn–Mn and other Zn-based aqueous batteries. The improvement in charge capability is attributed to stable electrolyte pH and activated Mn^2+^ deposition reaction by ZSH. And the improved discharge efficiency originates from the highly active Zn_2_Mn_3_O_8_ H_2_O nanorods array that are deposited during the charging process. The ZnO additive to the electrolyte consumes H^+^ ions and maintains the electrolyte pH value (pH ~ 6.4) by creating a neutral environment to facilitate Mn^2+^ deposition during charging. Concurrently, we established that the ZSH generation occurring in the ZnO gel-like electrolyte also lowers the Mn^2+^ deposition overpotential, due to a basic characteristic of ZSH. Moreover, the large amount of ZSH generated during charging promotes the formation of an electrolyte gel with lower water content that consequently reduces the gas evolution during the charging process. We have provided a new strategy to boost the cathode performance of electrolytic aqueous Zn–Mn battery, which may lay the foundation to flow-stack battery for stationary energy storage.

## Supplementary Information

Below is the link to the electronic supplementary material.

Supplementary file1 (PDF 2977 kb)
