# Supplementary material for: ZnO Additive Boosts Charging Speed and Cycling Stability of Electrolytic Zn–Mn Batteries
Source: Nanomicro Lett. 2024 Jan 4;16:74. doi: 10.1007/s40820-023-01296-y (PMC10767122; doi:10.1007/s40820-023-01296-y)
Supplement: Supplementary file 1 — Supplementary file1 (PDF 2977 kb) [file 40820_2023_1296_MOESM1_ESM.pdf]

Supporting Information for

ZnO Additive Boosts Charging Speed and Cycling Stability of Electrolytic Zn-Mn Batteries

Jin Wu[1], Yang Tang[1], Haohang Xu[1], Guandie Ma[1], Jinhong Jiang[1], Changpeng Xian[1], Maowen Xu[1], Shu-Juan Bao[1,*] and Hao Chen[1,*]

[1]Institute for Clean energy & Advanced Materials, School of Materials and Energy, Southwest University, Chongqing 400715, P. R. China

*Corresponding authors. E-mail: baoshj@swu.edu.cn (Shu-Juan Bao); haochenswu@163.com (Hao Chen)

S1 Experimental Section

S1.1 Materials

Zinc sulfate heptahydrate ($ZnSO_4{\cdot}7H_2O$, 99.5%), manganese sulfate monohydrate ($MnSO_4{\cdot}H_2O$, 99.0%) and zinc oxide (ZnO, 99%) were purchased from Aladdin, China. Carbon nanotubes film was purchased from Hongyun Technology Co., Ltd., Shandong, China. All reagents and materials were purchased commercially without further purification.

To prepare the ZnO gel-like electrolyte, 0.5, 1, 1.5, 2 and 2.5 grams of ZnO were added to a 10 mL solution consisting of 1 M $ZnSO_4$ and 2 M $MnSO_4$. The mixture was then stirred for 10 minutes to obtain the ZnO gel-like electrolyte. If not specified otherwise, the concentration of ZnO in the ZnO-gel-like electrolyte is 0.2 g mL.

S1.2 Battery Assembly

In our research, we utilized a transparent glass cell to create a visually transparent electrolytic Zn-Mn battery. Carbon nanotubes film served as the cathode substrate, while polished Zn foil (thickness approximately 0.2 mm) and 1 M $ZnSO_4$ + 2 M $MnSO_4$ aqueous solution were used as the anode and electrolyte, respectively. We added 0.66% (w/w) bromocresol green (dissolved in 1% (w/w) ethanol) to the electrolyte to investigate the pH change in situ. The bromocresol green undergoes a color change between pH 3.8 and 5.5, and the pH value of the standard electrolyte (1 M $ZnSO_4$ + 2 M $MnSO_4$) is 4.6.

To perform the gas production measurements on the electrolytic Zn-Mn batteries, the soft packed battery with a gas guide tube was assembled. The cathode consisted of a CNTs film with an area of 12 cm^{-2}, while the anode was made of Zn metal foil with the same area. The Whatman fiberglass was used as the separator between the two electrodes. The CNTs film was firmly pressed onto the steel foil on the cathode side.

Finally, we wrapped the battery with polyvinyl chloride film and sealed it using hot melt adhesive to ensure its integrity.

To perform electrochemical measurements on the electrolytic Zn-Mn batteries, we employed CR2032 coin-type cells. We used glass fiber (Whatman, GF/D) as the separator, carbon nanotubes films (thickness approximately 0.06 mm, area 1.13 cm^{-2}, weight 1.5 mg) as the cathode substrate, and polished Zn foil (thickness approximately 0.2 mm, area 1.33 cm^{-2}) as the anode electrode. We assembled different CR2032 coin-type electrolytic Zn-Mn batteries with varying electrolytes to conduct different electrolyte performance tests. The volume of electrolyte used in each CR2032 cell was 100 μL.

The square battery utilized Glass fiber as the separator (Whatman, GF/D), with carbon nanotubes films (thickness ~ 0.06 mm, 20 cm^{-2}) serving as the cathode substrate and polished Zn foil (thickness ~ 0.05 mm, 20 cm^{-2}) acting as the anode electrode. A titanium foil was employed as the current collector, and it leads out the tabs. To serve as the electrolyte, a mixture of 1 M $ZnSO_4$ + 2 M $MnSO_4$, and 0.25 g mL^{-1} ZnO was utilized. In assembling the pouch shell battery, four slices of CNT cathode were stacked with five slices of Zn metal anode in a forked laminated layout.

The two pouch cells were then assembled into a square shell battery. For the monolithic pouch battery, glass fiber was used as the separator (Whatman, GF/D), with carbon nanotubes membrane (thickness ~ 0.06 mm, 10 cm^{-2}) serving as the cathode electrode while zinc sheet (thickness ~ 0.2 mm, 10 cm^{-2}) was used as the anode electrode. The pouch battery was sealed, and during charging, N_2 was used as the carrier gas to evaluate the generation of gas.

S1.3 Electrochemical Measurements

The chronoamperometric charge and galvanostatic discharge test of the assembled batteries were carried out on the LAND CT2001 test system (Wuhan LAND electronics Co., Ltd, China). Cyclic voltammetry (CV) tests were performed on the CHI760e electrochemical workstation (Chenhua Instrument Company, Shanghai, China). In this study, the test methods of constant voltage charging and constant current discharge were adopted. The voltages in the test are all voltages relative to vs Zn/Zn^{2+}.

S1.4 Material Characterization

The phase structure of the electrode materials was determined using X-ray diffraction (XRD), specifically the Shimadzu-7000 model. For observation of morphology, microstructure, and composition, field emission scanning electron microscopy (FESEM) equipped with an energy dispersive spectrometer (EDS) was utilized, specifically the JSM-7800F model. The Raman spectra of the samples were recorded using the LabRAM HR Evolution (Horiba) with a laser excitation at 532 nm. To investigate the surface chemical composition and valence state, X-ray photoelectron spectroscopy (XPS) with the Escalab 250xi model was used. Before testing, all electrodes were washed with deionized water to remove any residual electrolyte. The Mn K-edge X-ray absorption spectra were conducted in fluorescence mode at the BL14W1 beamline of

the Shanghai Synchrotron Radiation Facility, China, operating at 3.5 GeV with maximum injection currents of 230 mA. To reduce the harmonic component of the monochrome beam, a double-crystal monochromator equipped with a Si (111) crystal was used to monochromatize the synchrotron beam. Reference samples, specifically Mn_3O_4 standard and MnO_2 standard, were used in the experiment. To detect the types of gases produced during battery cycling, meteorological mass spectrometers (GSD320 Omnistar) were utilized with N_2 as the carrier gas. The Optima 8000 model of the inductively coupled plasma optical emission spectrometer (ICP-OES) was applied to measure the mass fraction of residual iron catalysts in carbon nanotubes membranes.

S1.5 Methods of Drawing the Pourbaix Diagram

The Pourbaix diagram in the **Fig. 2A** is get from an Open-Source calculation system of THE MATERIALS PROJECT (https://next-gen.materialsproject.org/). THE MATERIALS PROJECT is a multi-institution, multi-national effort to compute the properties of all inorganic materials and provide the data and associated analysis algorithms for every materials researcher free of charge. In this system, the Pourbaix Diagram can be obtained by inputting the composition of the electrolyte.

S1.6 ZHS Assisted Mn^{2+} Deposition-dissolution Reaction

Charge Process:

$$Zn_4SO_4(OH)_6{\cdot}nH_2O + Mn^{2+} \rightarrow Zn_xMnO_2{\cdot}H_2O + 4H^+ + SO_4^{2-} + 2e^- \quad (S1)$$

Discharge process:

$$Zn_xMnO_2{\cdot}H_2O + 4H^+ + 2e^- \rightarrow Mn^{2+} + Zn^{2+} + 3H_2O \quad (S2)$$

$$4Zn^{2+} + SO_4^{2-} + 6OH^- + nH_2O \rightarrow Zn_4SO_4(OH)_6{\cdot}nH_2O \quad (S3)$$

S1.7 Formation Reaction of ZHS in the ZnO Gel-like Electrolyte

$$H_2O \rightarrow H^+ + OH^- \quad (S4)$$

$$ZnO + 2H^+ \rightarrow Zn^{2+} + H_2O \quad (S5)$$

$$4Zn^{2+} + SO_4^{2-} + 6OH^- + 4H_2O \rightarrow Zn_4SO_4(OH)_6{\cdot}4H_2O \quad (S6)$$

During the deposition process of ZSH, the Zn $(H_2O)_6^{2+}$ first deprotonation step to the intermediate amphoteric $[Zn(H_2O)_5(OH)]^+$, and then further lead to neutral double hydroxide $Zn(H_2O)_4(OH)_2$ species. The presence of co-precipitated SO_4^{2-} can slowly convert into the crystalline of $Zn(H_2O)_4(OH)_2$ to form $Zn_4SO_4(OH)_6{\cdot}4H_2O$ (**Fig. S5**) [S1]. Thus, in order to better query the standard equilibrium constant, the **Equation S6** was equivalently substituted, and shown in as follow:

$$3Zn^{2+} + 6OH^- \rightarrow 3Zn(OH)_2 \quad (S7)$$

Given that the $K^{\theta}sp$ of $Zn(OH)_2$ is 1×10^{-17}, the $C(Zn^{2+})$ is 1M. Thus, the start deposition pH of $Zn_4SO_4(OH)_6{\cdot}4H_2O$ is around 5.5 in 1M $ZnSO_4$. At the same time, according the references [S2, S3], the starting pH of ZnO dissolution reaction is also around 5.5. Thus, when ZnO is added to the solution of 1M $ZnSO_4$ + 2 M $MnSO_4$ (pH is around 4.6), the **Eqs. S5** and **S6** is occurs rapidly until all the ZnO is consumed.

S2 Supplementary Figures and Tables

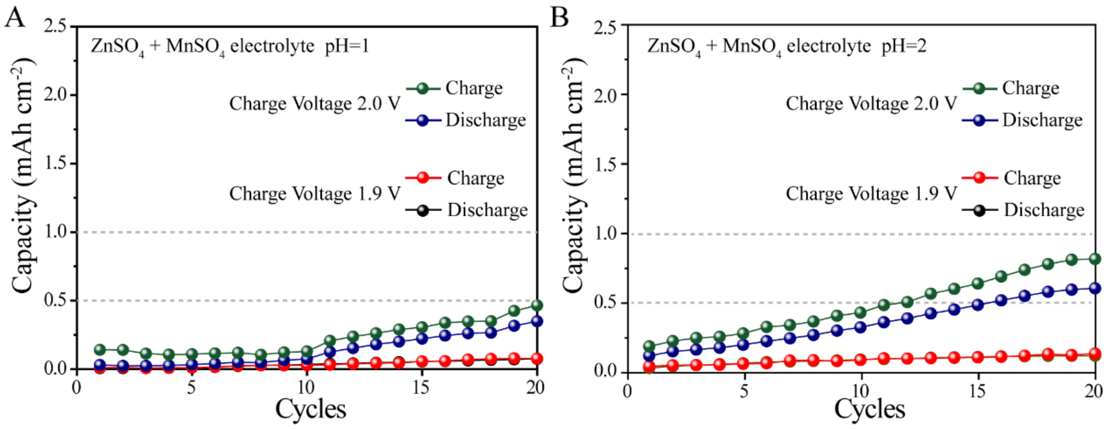

Fig. S1 Charge surface area capacity and discharge surface area capacity during of the button electrolytic Zn-Mn batteries during the initial 20 cycles in different conventional aqueous electrolyte. (A) the pH of electrolyte is 1, (B) the pH of electrolyte is 2. The constant voltage charging time is 30min, and the constant current discharge current is 0.5 mA cm^{-2}

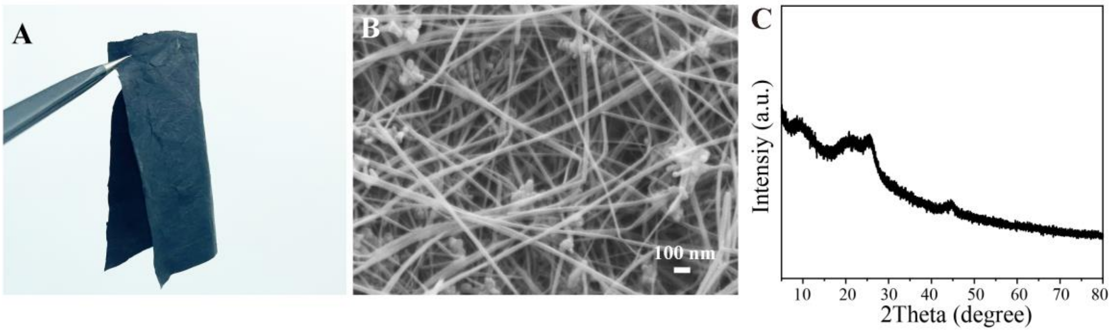

Fig. S2 (**A**) Optical photograph, (**B**) FESEM image and (**C**) XRD pattern of the carbon nanotubes film (CNTs)

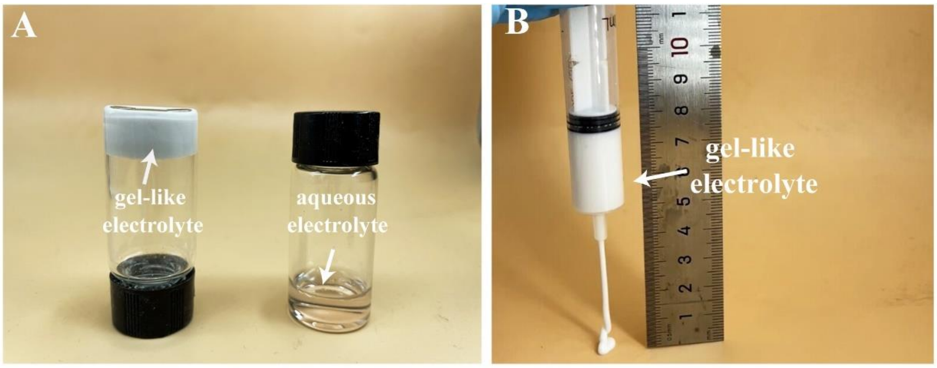

Fig. S3 (**A**) Optical photograph of ZnO gel-like electrolyte and conventional aqueous electrolyte. (**B**) Optical photograph shows that the ZnO gel-like electrolyte could be extruded from the syringe and self-support

Table S1 The ionic conductivity of different electrolyte

electrolyte	ionic conductivity (mS cm^{-1})
1 M $ZnSO_4$ + 2 M $MnSO_4$	34.985
1 M $ZnSO_4$ + 2 M $MnSO_4$ + 0.1g mL^{-1} ZnO	28.726
1 M $ZnSO_4$ + 2 M $MnSO_4$ + 0.15g mL^{-1} ZnO	26.829
1 M $ZnSO_4$ + 2 M $MnSO_4$ + 0.2g mL^{-1} ZnO	22.693
1 M $ZnSO_4$ + 2 M $MnSO_4$ + 0.25g mL^{-1} ZnO	22.152

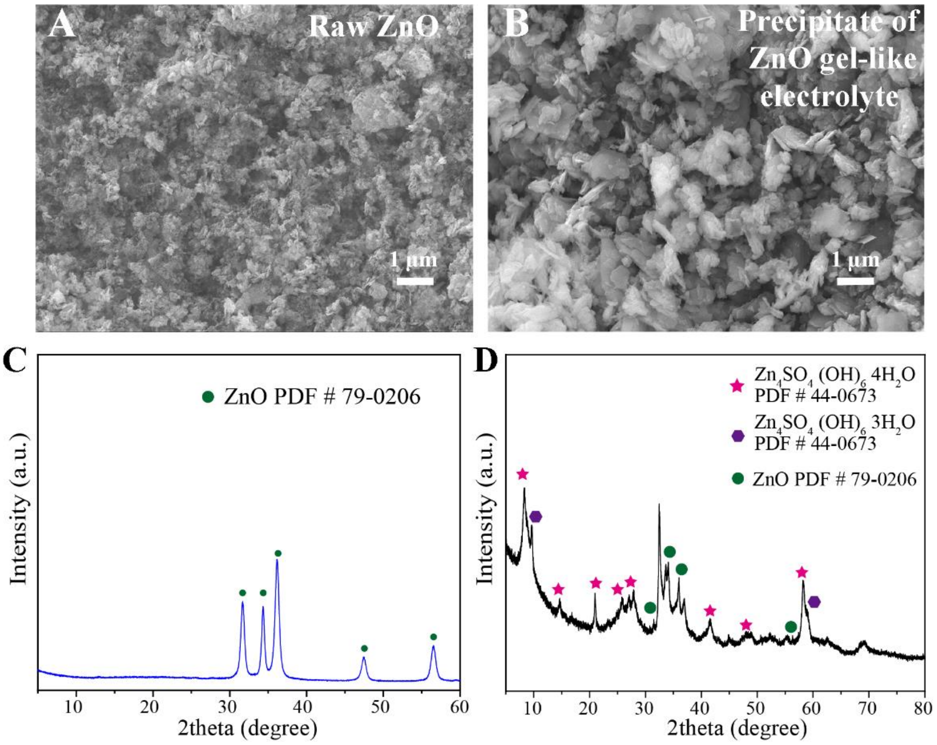

Fig. S4 FESEM images of (**A**) ZnO powder, (**B**) the ZnO gel-like electrolyte after drying. XRD pattern of (**C**) ZnO powder and (**D**) the gel-like electrolyte after washing and drying

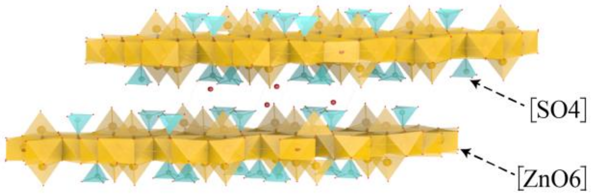

Fig. S5 The crystal structure of $Zn_4SO_4(OH)_6 \cdot 4H_2O$

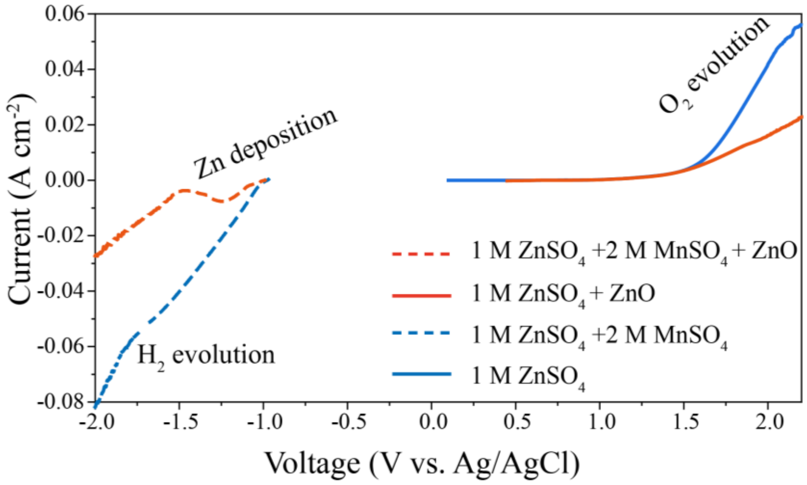

Fig. S6 The linear sweep voltammetry analysis at a scan rate of 1 mV s^{-1}. In the right part, the working electrode is CNTs film, counter electrode is Zn foil, and reference is Ag/AgCl electrode. For the left part, the working electrode is replaced with Zn foil, while the other electrodes remain unchanged. To avoid interference from MnO_2 deposition reaction to the exploration of OER, the electrolyte used in the right part does not contain Mn^{2+}

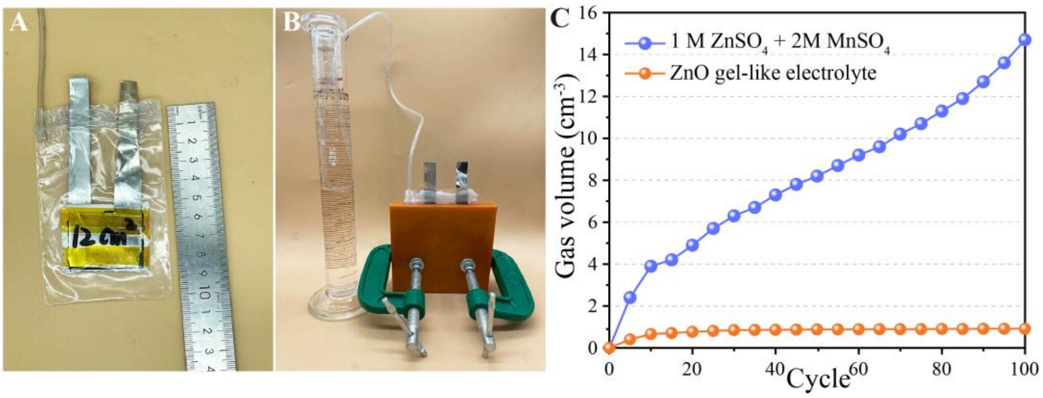

Fig. S7 Gas production test of electrolytic Zn-Mn batteries. (**A and B**) Prepared soft packed batteries and gas measurement device respectively. (**C**) The volume of gas produced during the battery cycling process. Chronoamperometric charge under 2.0 V *vs.* Zn/Zn^{2+} to 0.5 mAh cm^{-2} and discharge at 2 mA cm^{-2}

We collected the linear sweep voltammetry curves to analysis the HER and OER property during the first charge process (**Fig. S6**). Obviously, after introducing the

ZnO electrolyte addition, the HER and OER were suppressed. Specifically, the OER reaction current density is decreased, the HER overpotential is increased and current density is decreased.

To further investigate the OER and HER reaction during the cycling, the gas volume produced from the electrolytic Zn-Mn batteries with different electrolyte during the cycling were record. As shown in the **Fig. S7A**, a soft packed battery with a gas guide tube was prepared, the area of CNTs film cathode is 12 cm^{-2}, the Zn metal foil is anode and Whatman fiberglass is the separate. The gas volume measuring device is shown in the **Fig. S7B**. To ensure there is no residual gas within the soft packed battery, a fixture was used to secure the soft pack battery. When using ZnO gel-like electrolyte, the max gas produce volume of soft packed electrolytic Zn-Mn batteries is only 0.91 cm^{-3} after 100 cycling (chronoamperometric charge of 2.0 V *vs.* Zn/Zn^{2+} to 1.5 mAh cm^{-2}, discharge to 0.8 V *vs.* Zn/Zn^{2+} with 1 mA cm^{-2}). Under the same charge-discharge conditions, when without ZnO electrolyte additive, the gas produce volume is continually increased, and reach 14.7 cm^{-3} at 100th cycle. Obviously, the ZnO gel-like electrolyte greatly suppresses the gas generation in the electrolytic Zn-Mn batteries during the cycling.

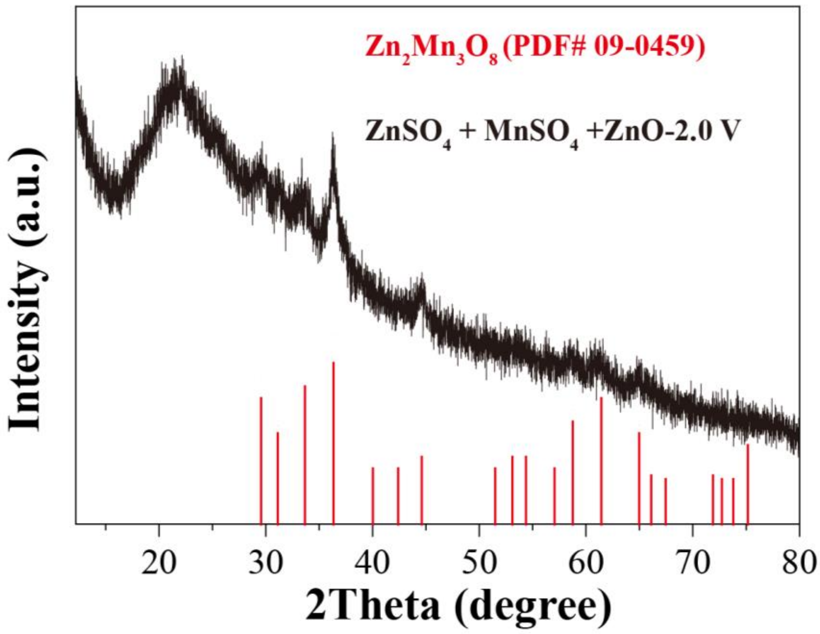

Fig. S8 Amplifying XRD pattern of CNTs cathode at the full charged state when using ZnO gel-like electrolyte as the electrolyte of batteries

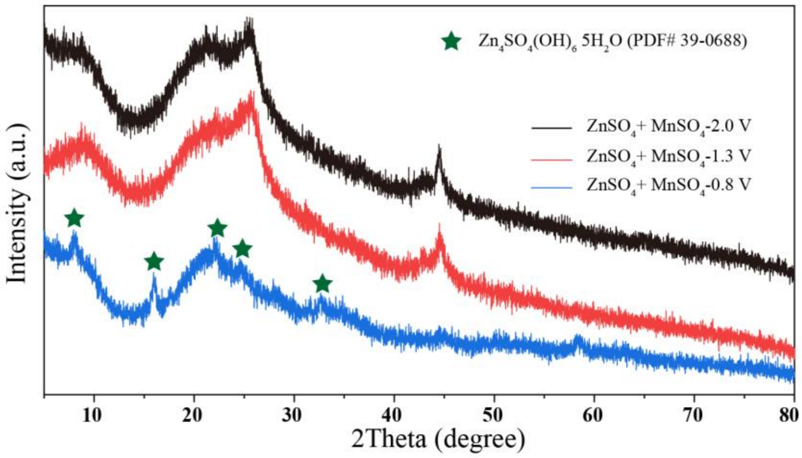

Fig. S9 Ex-situ XRD patterns of CNTs cathode with conventional aqueous electrolyte at different charge–discharge states. The charge capacity is 1.5 mAh cm^{-2}, and the charge voltage is 2 V *vs.* Zn/Zn^{2+}, and the discharged current is 0.1 mA cm^{-2}.

Table S2 The deposition mass of cathode after chronoamperometric charge of 2V *vs.* Zn/Zn^{2+} in different electrolytes

Charge time	ZnO gel-like electrolyte	Conventional aqueous electrolyte
300 s	1.9 mg	0.5 mg
1200 s	3.2 mg	1 mg
1800 s	3.7 mg	1.6 mg

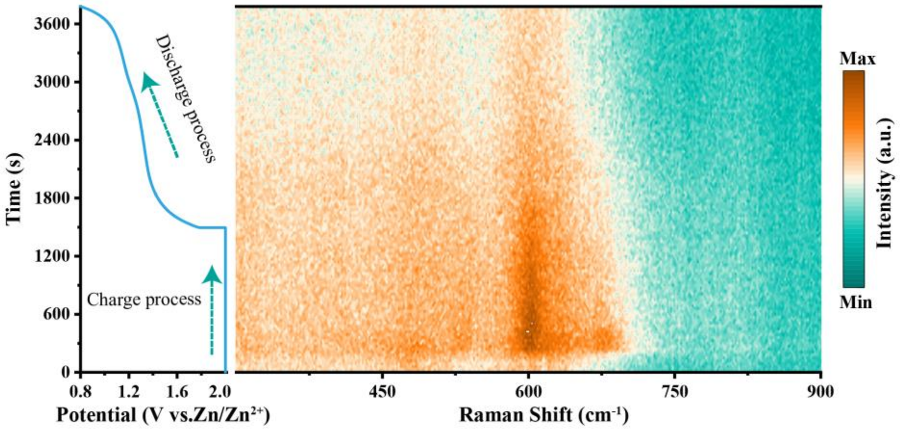

Fig. S10 Operando Raman spectra of the CNTs cathode with ZnO gel-like electrolyte.

Figure S10 illustrated operando Raman spectra of the CNTs cathode during the charge-discharge process with ZnO gel-like electrolyte. Initially, no observable peaks were detected in the fresh CNTs. However, after the completion of charging, three distinct Raman bands appeared at 502, 572, and 676 cm^{-1}, which correspond to the V4 (Mn–O) stretching vibration mode, stretching vibration of V3(Mn–O) in the basal plane of [MnO6] sheets and symmetric stretching vibration of V2 (Mn–O) in MnO6. This observation suggests the formation of layered manganese oxides. Upon discharging,

these bands gradually weakened and eventually disappeared, which is consistent with previous studies [4].

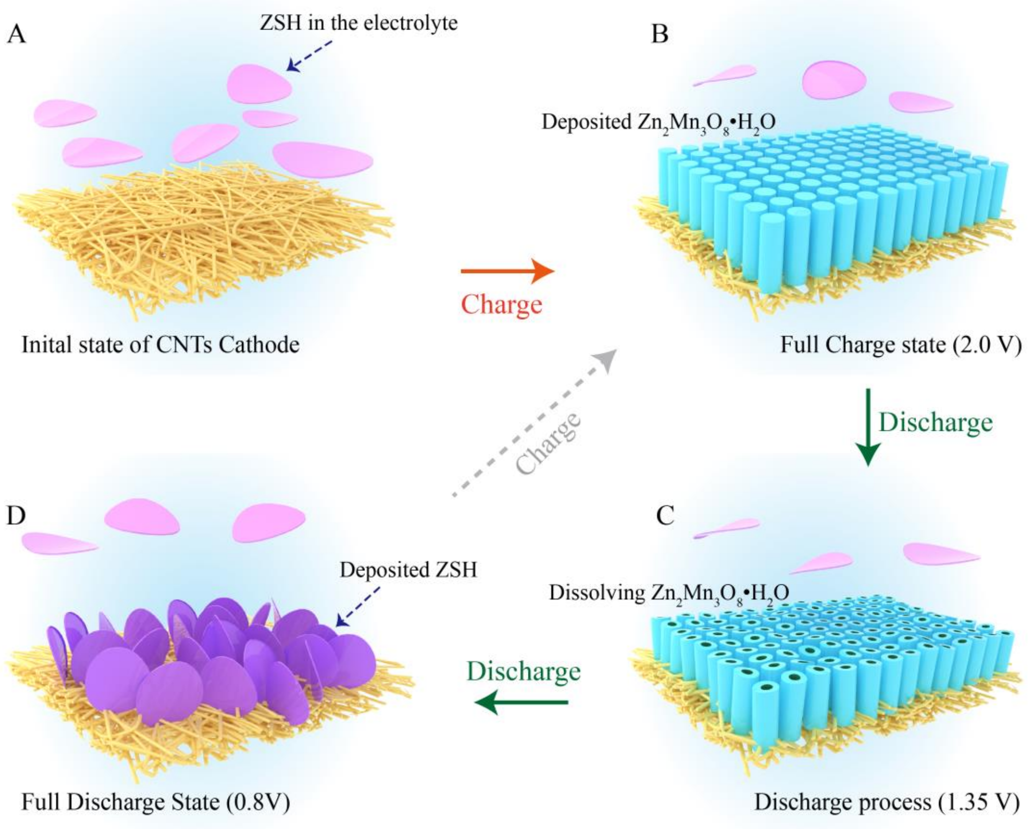

Fig. S11 Schematic illustrating the morphology and phase evolution of cathode of during the charging and discharging reactions

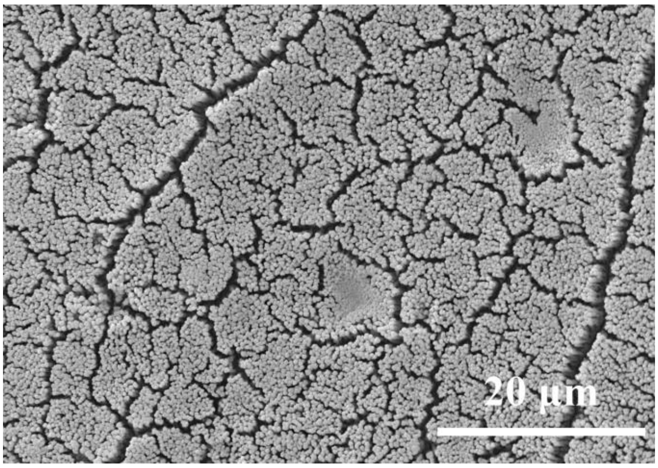

Fig. S12 A broader field SEM image of CNTs cathode after chronoamperometric charge process. (Chronoamperometric voltage of 2.0 V *vs.* Zn/Zn^{2+} to 1.5 mAh cm^{-2})

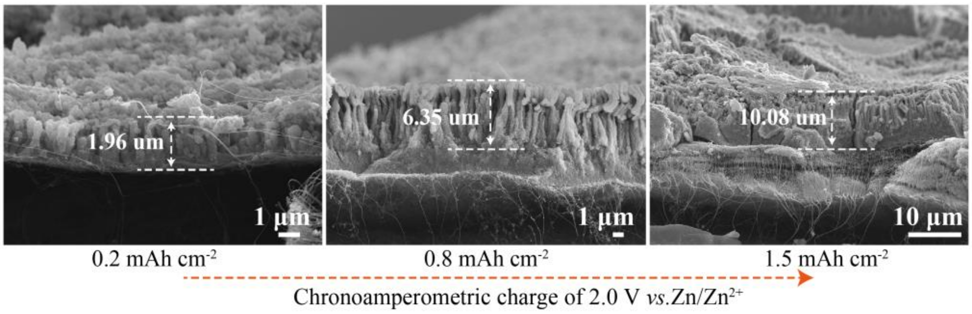

Fig. S13 The cross-section view SEM images of the CNTs cathode during the charge process

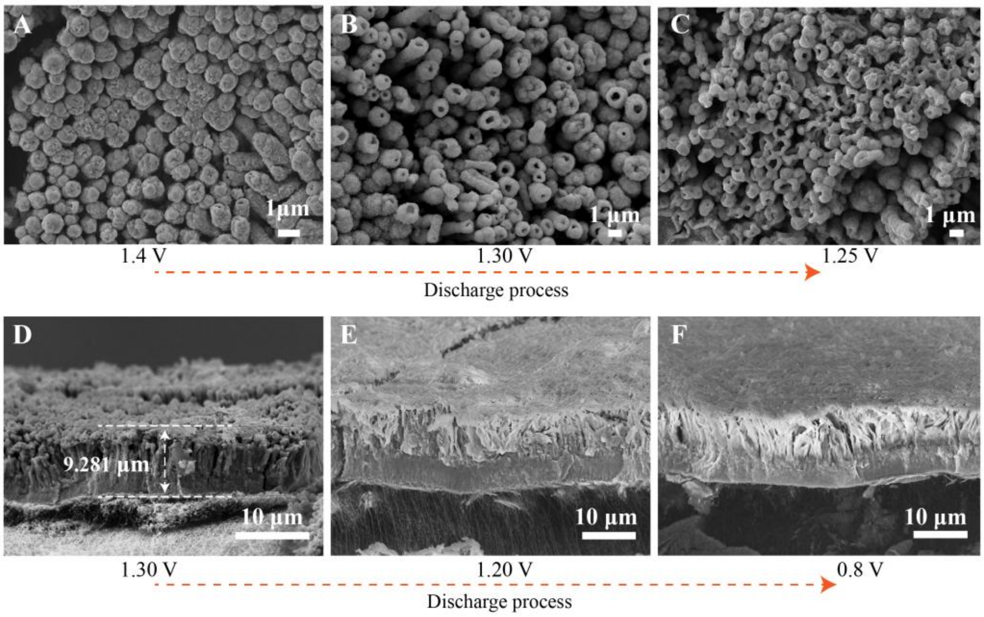

Fig. S14 (**A-C**) The SEM images of cathode surface during the discharge process. (**D-F**) The SEM images of cathode cross-section during the discharge process. (Chronoamperometric charge under 2.0 V *vs.* Zn/Zn^{2+} to 1.5 mAh cm^{-2} and discharge at 1 mA cm^{-2})

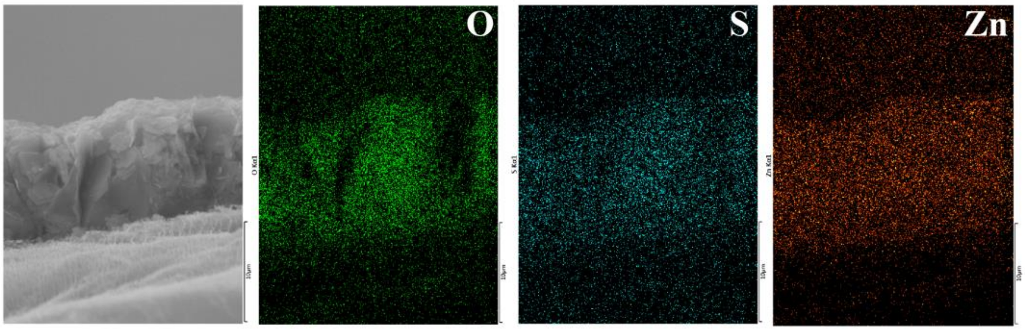

Fig. S15 The SEM-Energy Dispersive Spectrometer (EDS) mapping of CNTs cathode at discharge state of 0.8 V *vs.* Zn/Zn^{2+}. (Chronoamperometric charge under 2.0 V *vs.* Zn/Zn^{2+} to 1.5 mAh cm^{-2} and discharge at 1 mA cm^{-2})

A schematic diagram (**Fig. S11**) was presented to illustrating the evolution process of phase and structure on the cathode surface during the charge-discharge process. The more detail about the morphology evolution of cathode during the charge-discharge process was collected and shown in the **Figs. S12-S15.**

As shown in the **Fig. S11B**, due to the presence of ZSH in the electrolyte, the $Zn_2Mn_3O_8{\cdot}H_2O$ nanorods were deposited on the electrode surface during the charge process. The reaction mechanism can be written as follows:

$$Zn_4SO_4(OH)_6{\cdot}nH_2O\ \text{(electrolyte)} + 6Mn^{2+} \rightarrow 2Zn_2Mn_3O_8 \cdot H_2O + H^{+} + SO_4^{2-} + 12e^{-}$$

The growth process of $Zn_2Mn_3O_8{\cdot}H_2O$ nanorods was shown in the **Fig. S13**. With the increased charge capacity, the length of $Zn_2Mn_3O_8{\cdot}H_2O$ nanorods continuously grows, eventually reaching 10.08 μm when it attains 0.8 mAh cm^{-2}.

As illustrated in **Fig. S11C**, during the discharge process, the deposited $Zn_2Mn_3O_8{\cdot}H_2O$ dissolve slowly through a process of hollowing. The reaction mechanism can be written as follows:

$$Zn_2Mn_3O_8{\cdot}H_2O + 7H_2O + 6e^{-} \rightarrow 3Mn^{2+} + 2Zn^{2+} + 16OH^{-}$$

The dissolution process of $Zn_2Mn_3O_8{\cdot}H_2O$ nanorods was shown in the **Fig. S14A**. When discharge to 1.4 V *vs.* Zn/Zn^{2+}, small downward depressions and wrinkles start to appear at the cross-section of $Zn_2Mn_3O_8{\cdot}H_2O$ nanorods. And then, these concave $Zn_2Mn_3O_8{\cdot}H_2O$ nanorods transformed into hollow structure at 1.30 V *vs.* Zn/Zn^{2+} (**Fig. S14B**). With further discharge, the hollow nanorod structure further collapse and dissolve at 1.25 V *vs.* Zn/Zn^{2+} (**Fig. S14C**). From the **Fig. S14D**, it is interesting to find that the thickness of the $Zn_2Mn_3O_8{\cdot}H_2O$ nanorods layer does not show significant changes before discharging to 1.3V *vs.* Zn/Zn^{2+}, which demonstrated that the main contribution to the capacity before discharge to 1.3 *vs.* Zn/Zn^{2+} comes from the hollowing of the $Zn_2Mn_3O_8{\cdot}H_2O$ nanorods.

At the discharge state of 1.2 *vs.* Zn/Zn^{2+}, a lot of flakes was deposited on the cathode surface, and eventually occupied the entire surface of the cathode at the 0.8 V *vs.* Zn/Zn^{2+}. According to the XRD patterns (**Fig. 3A**) and SEM-EDS mapping (**Fig. S15**), the deposited flakes on the cathode surface is $Zn_4SO_4(OH)_6{\cdot}nH_2O$ (ZSH), and the deposition mechanism can be written as follow:

$$4Zn^{2+} + SO_4^{2-} + 6OH^{-} + nH_2O \rightarrow Zn_4SO_4(OH)_6{\cdot}nH_2O\ \text{(cathode surface)}$$

The OH^{-} originate from the dissolution process of $Zn_2Mn_3O_8{\cdot}H_2O$. The deposition of ZSH was also illustrated in the **Fig. S11D**.

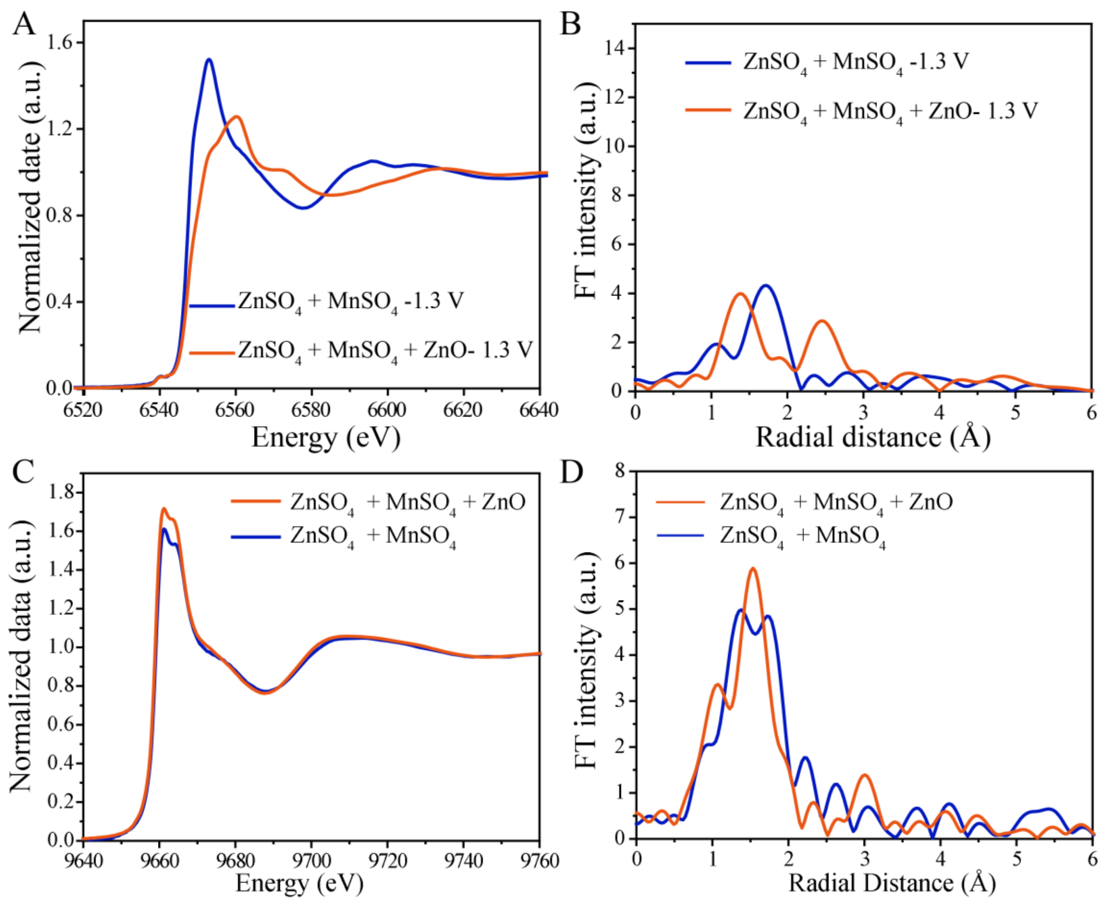

Fig. S16 (**A**) Normalized Mn K-edge XANES spectra and (**B**) Corresponding EXAFS spectra in r-space of the CNTs cathode in ZnO gel-like electrolyte at discharge state of 1.3 V *vs.* Zn/Zn^{2+}. (**C**) Normalized Zn K-edge XANES spectra and (**D**) EXAFS spectra in r-space of the CNTs cathode in conventional aqueous electrolyte and ZnO gel-like electrolyte (30 min chronoamperometric charge of 2 V *vs.* Zn/Zn^{2+} and discharged to 1.3 V *vs.* Zn/Zn^{2+} under 0.1mAh cm^{-2})

Figure S16A, B demonstrated that the valence state of Mn on the cathode decreased following discharge, regardless of electrolyte type. Specifically, in ZnO gel-like electrolytes, the average valence of manganese in manganese oxides drops from approximately 4 to 3.5. However, in the conventional aqueous electrolyte, the valence state of manganese hardly changes at 1.3 V *vs* Zn/Zn^{2+}, remaining around 3.

We collected the XAFS of Zn K-edge in this sample (**Fig. S16C, D**), the presence of Zn^{2+} in manganese oxides deposited in conventional aqueous electrolyte and gel-like electrolyte. These indicate that the deposited products are all zinc-manganese oxides, but deposited in the form of $Zn_{\alpha}MnO_x$ (Z < 4 for Mn^{z+}), and deposited in the form of $Zn_2Mn_3O_8$ (Z = 4 for Mn^{z+}) after full charge in gel-like electrolyte.

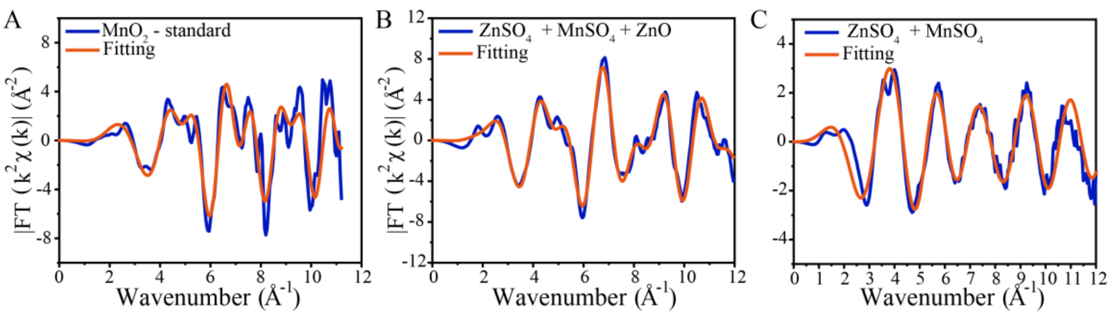

Fig. S17 FT of the k^3-weighted EXAFS spectrum and fit real components at of the (**A**) MnO_2 standard, CNTs cathode after full charging in (**B**) conventional aqueous electrolyte and (**C**) ZnO gel-like electrolyte after 30 min chronoamperometric charge of 2 V *vs.* Zn/Zn^{2+}

Table S3 Structural parameters of the sample obtained from the XAFS fitting

Sample	Theoretical FEFF	Shell	Scattering path	S_0^2	N	ΔE_0(eV)	R(Å)	σ^2	R-factor
MnO_2 (Standard)	MnO_2	1st	Mn-O	1.879	6	-2.296	1.84(0.018)	0.00184	0.0232
		2nd	Mn-Mn	1.253	4		2.86(0.033)	0.00644	
		2nd	Mn-Mn	1.253	4		3.42(0.032)	0.00028	
Conventional aqueous electrolyte	$ZnMn_2O_4$	1st	Mn-O	2.262	4	-9.605	1.91(0.029)	0.00921	0.0064
	MnO	2nd	Mn-O	3.393	6		2.14(0.020)	0.00474	
	$ZnMn_2O_4$	3rd	Mn-Mn	1.131	2		2.74(0.041)	0.01927	
ZnO Gel-like electrolyte	$Zn_2Mn_3O_8$	1st	Mn-O	2.670	1	0.997	1.89(0.006)	0.00193	0.0041
		2nd	Mn-Mn	2.525	2		2.93(0.016)	0.00598	
		3rd	Mn-Zn	5.340	2		3.30(0.053)	0.02242	

S_0^2: amplitude reduction factor; N: coordination number; ΔE_0: shift in edge energy; R(Å): atomic distance; σ^2: Debye–Waller factor. The bold numbers were obtained by LCF (weight fraction), reference EXAFS fitting (S_0^2), or ideal values based on stoichiometry (N), and were fixed during these fitting operations.

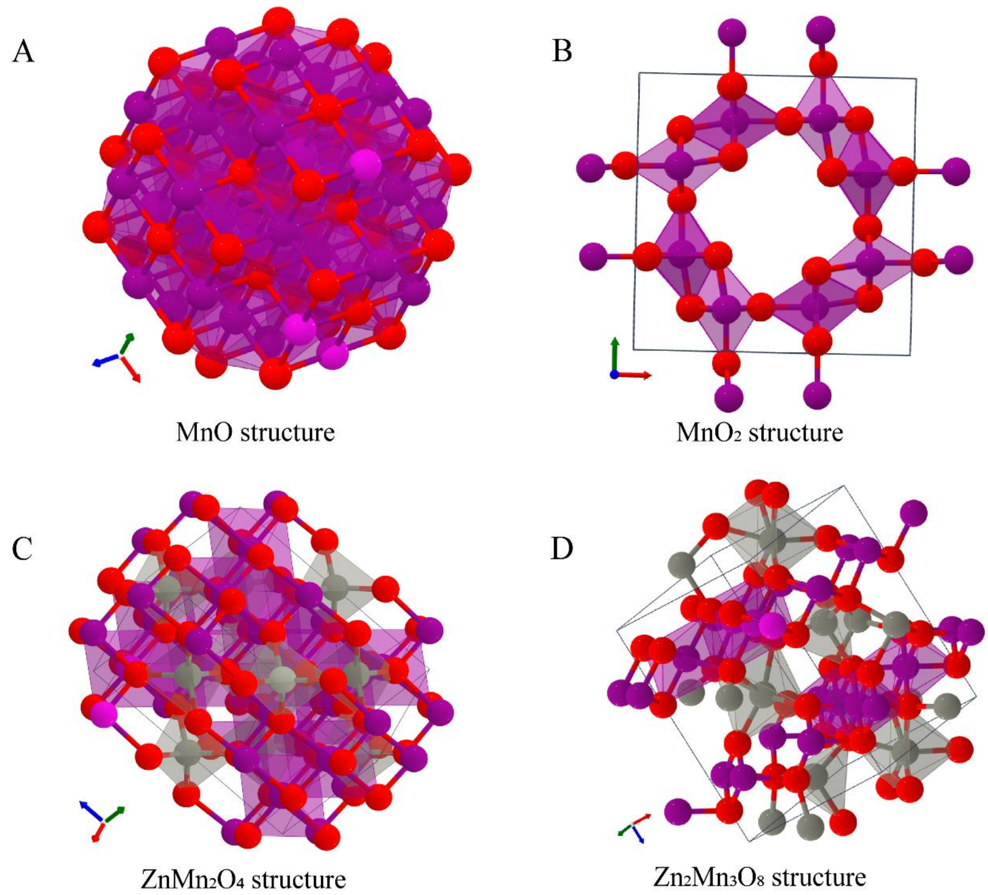

Fig. S18 Theoretical structures used to perform FEFF calculations during EXAFS fitting

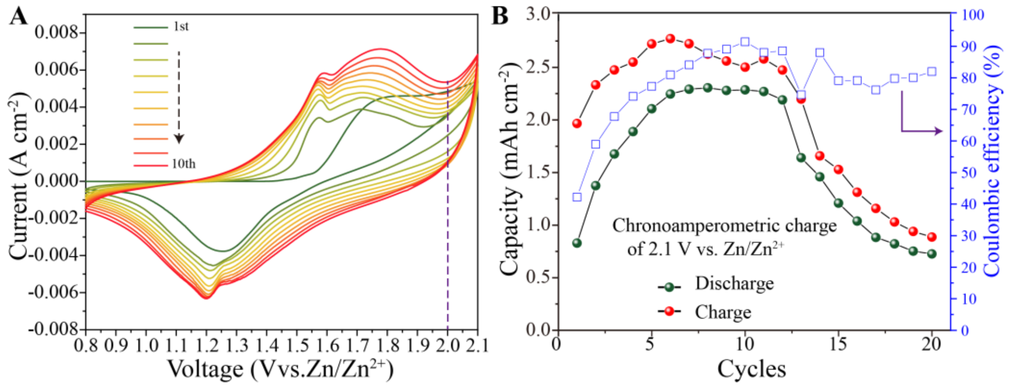

Fig. S19 (**A**) Cyclic voltammetry (CV) curves generated using a scanning rate of 1 mV s^{-1} for electrolytic Zn-Mn coin cell batteries with ZnO gel-like electrolyte. (**B**) The Charge and discharge capacities of button electrolytic Zn-Mn batteries with ZnO gel-like electrolyte during the initial 20 cycles at a chronoamperometric charge under 2.0 V *vs.* Zn/Zn^{2+} for 30 min.

During the deposition process of Mn^{2+}, the deposition overpotential of MnO_2 will gradually increase as the Mn^{2+} ions on the electrode surface are consumed. In the **Fig. 5A**, the tested electrolytic Zn-Mn batteries is assembled by using CR2032 coin-type

cell mold, an no additional electrolyte diffusion measures. Thus, the limited diffusion behavior of Mn^{2+} will cause the overpotential of the deposition reaction to continuously increase. As shown in the **Fig. S19A,** the CV peaks at 1.8 V *vs.* Zn/Zn^{2+} is very wide, and the current density is 5.3 mA cm^{-2} at 2.0 V *vs.* Zn/Zn^{2+} (The max current density is 0.7 mA cm^{-2} at 1.8 V). Thus, because of the limited diffusion behaviors of Mn^{2+}, the charge capacity can be significantly increased with increased charge voltage from 1.9 to 2.0 V *vs.* Zn/Zn^{2+}.

In theory, if the charging voltage is adjusted to 2.1 *vs.* Zn/Zn^{2+}, the higher charge capacity of electrolytic Zn-Mn batteries will be achieved. However, as shown in **Fig. S19A**, the detrimental OER reactions start to take place in the button cell as the voltage gradually increases to 2.1 V *vs.* Zn/Zn^{2+}. In **Fig. S19B**, when the chronoamperometric charge voltage is 2.1 V *vs.* Zn/Zn^{2+}, the charge capacity can reach 2.76 mAh cm^{-2} at the 6th cycles. However, the charge capacity begins to continuously decrease from the 7th cycle. Thus, in this works, we set the chronoamperometric charge voltage of the electrolytic Zn-Mn battery is 2.0 V *vs.* Zn/Zn^{2+}.

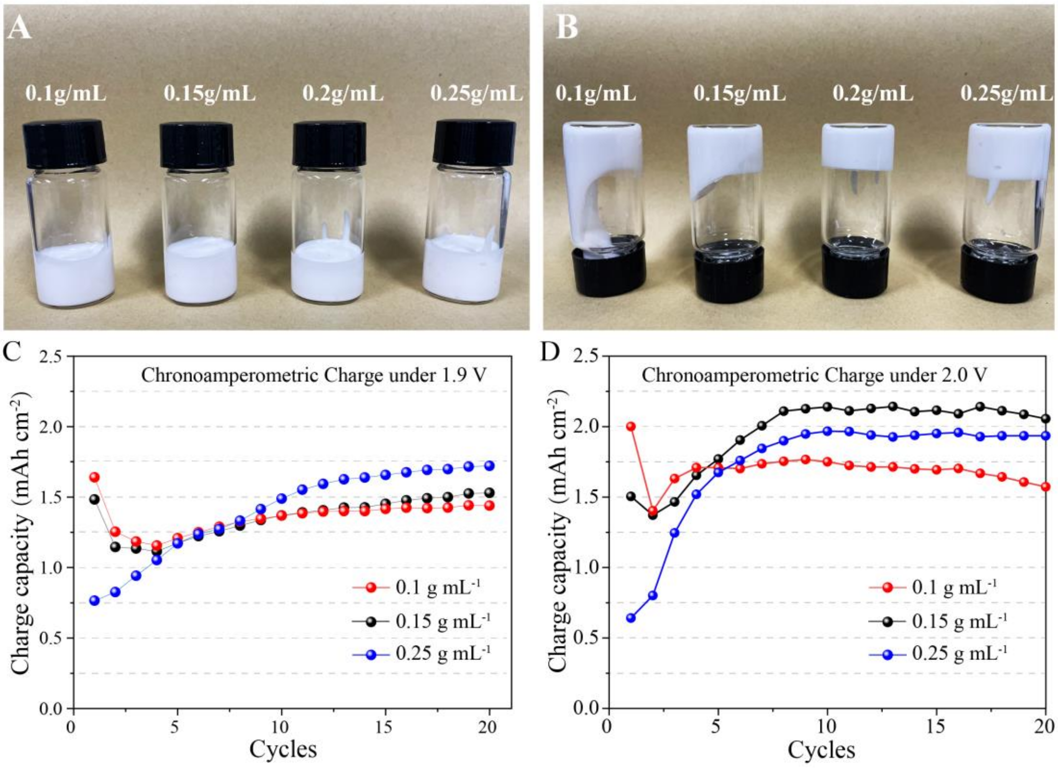

Fig. S20 The Digital image of ZnO-gel-like electrolyte with different with different amounts of ZnO. (**A**) upright and (**B**) upside down bottle. Charge capacities of button cell electrolytic Zn-Mn batteries during initial 20 cycles in ZnO gel-like electrolyte with different amount of ZnO addition. (**C**) chronoamperometric charge for 30 min under (A) 1.9 V *vs.* Zn/Zn^{2+} and (**D**) 2.0 V *vs.* Zn/Zn^{2+}. The discharge current is 1 mA cm^{-2}.

As shown in the **Fig. S20**, the prepared ZnO gel-like electrolyte with different amount ZnO addition all exhibit a uniformly gel-like appearance (**Fig. S20A**). By inverting the glass vial (**Fig. S20B**), we can observe that the liquidity of the electrolyte

tends to decrease as the amount of ZnO increases.

The **Fig. S20C, D** exhibit the charge capacities during initial 20 cycles of electrolytic Zn-Mn batteries with the different amount of ZnO addition (including 0.1, 0.15 and 0.25 g mL^{-1}). Obviously, compare to conventional aqueous electrolyte (**Fig. 5A**), the prepared ZnO gel-like electrolyte with the ZnO concentration of 0.1, 0.15 and 0.25 g mL^{-1} all can significantly improve the charging capacity of batteries. Under 30 min chronoamperometric charge voltage of 1.9 V *vs.* Zn/Zn^{2+} and 20 cycles, the charge capacities are 1.43, 1.53 and 1.72 mAh cm^{-2} in 0.1, and 0.15 and 0.25 g mL^{-1} respectively. When the charging voltage is increased to 2.0 V *vs.* Zn/Zn^{2+}, the charge capacities is increased to 1.57, 1.95 and 2.05 mAh cm^{-2} in 0.1, 0.25 and 0.15 g mL^{-1} respectively. Obviously, the electrolyte with an addition of 2 g mL^{-1} of ZnO exhibits superior ability in enhancing battery charging performance (1.74 and 2.48 mAh cm^{-2} under chronoamperometric charge voltage of 1.9 and 2.0 V vs Zn/Zn^{2+}) than other ZnO concentration, which may be attributed to the equilibrium between the free water and the amount of zinc oxide added in the electrolyte

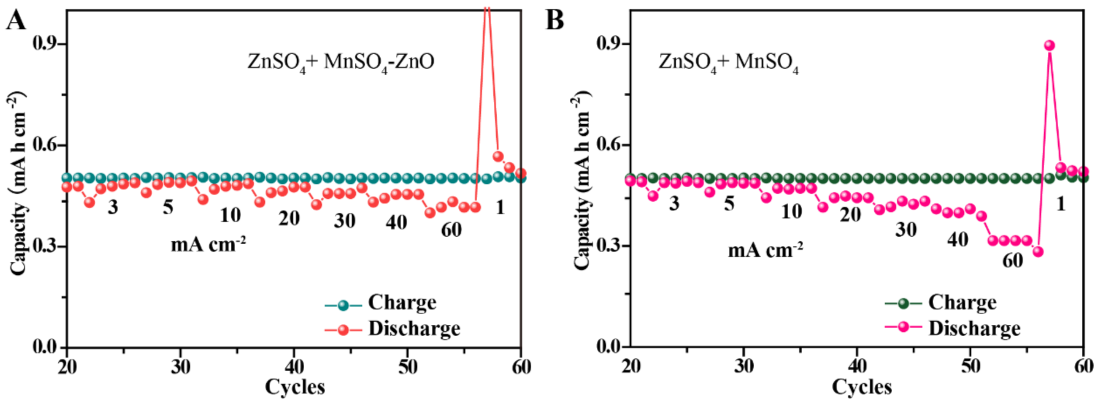

Fig. S21. Rate performance of the electrolytic Zn-Mn button batteries in (**A**) ZnO gel-like electrolyte and (**B**) conventional aqueous electrolyte

Figure S21 demonstrated that the use of ZnO gel-like electrolyte in electrolytic Zn-Mn batteries results in superior discharge rate performance compared to the use of conventional aqueous electrolyte. Specifically, at a discharge current of 60 mA cm^{-2}, the discharge capacity is 0.44 and 0.32 mAh cm^{-2} when using ZnO gel-like electrolyte and conventional aqueous electrolyte, respectively. The experiment involved charging at a constant voltage of 2V *vs.* Zn/Zn^{2+} to 0.5 mAh cm^{-2} and discharging to 0.8 V *vs.* Zn/Zn^{2+} with a constant current density. The addition of ZnO has significantly improved the rate performance of the battery.

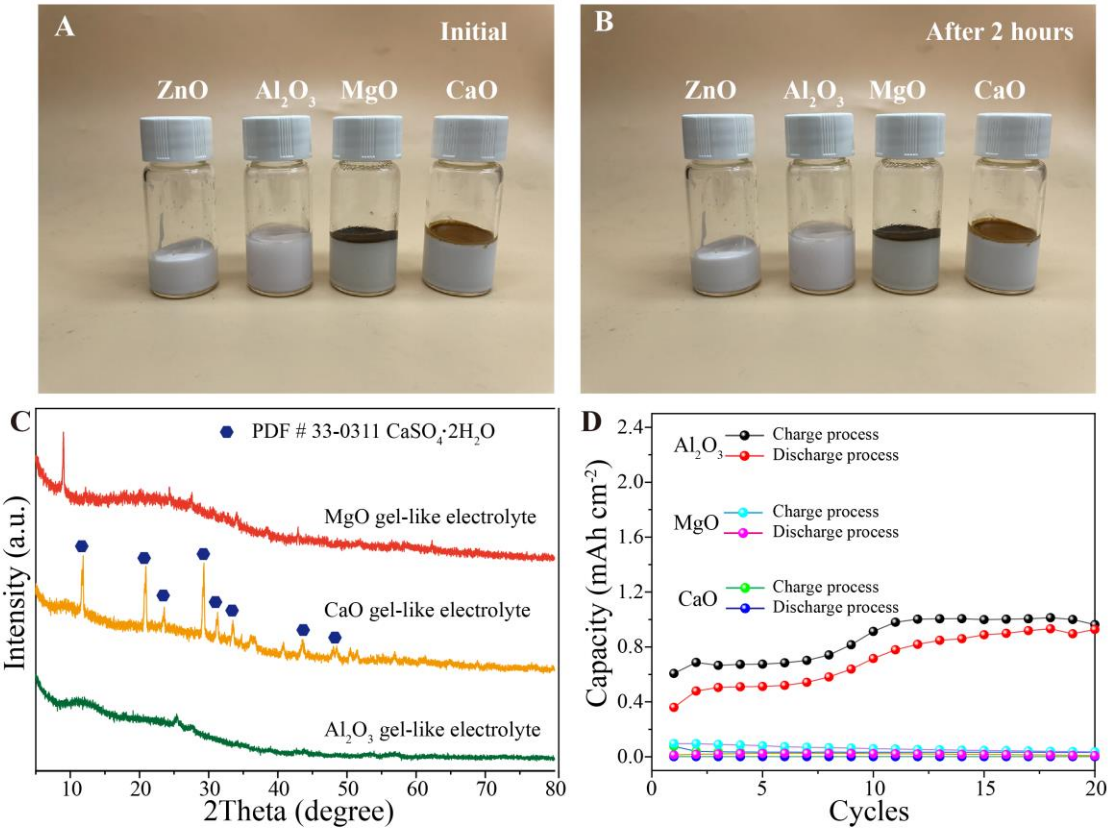

Fig. S22 (**A, B**) The digital photos of as-prepared 1 M $ZnSO_4$ + 2M $MnSO_4$ electrolytes with ZnO, Al_2O_3, MgO and CaO as the additive. (**C**) The XRD pattern of insoluble substance of the Al_2O_3, MgO and CaO gel-like electrolyte. (**D**) The charge-discharge capacities of different electrolyte during the initial 20 cycles. Chronoamperometric charge under 2.0 V *vs.* Zn/Zn^{2+} for 30 min, discharge at 1 mA cm^{-2} to 0.8 V *vs.* Zn/Zn^{2+}.

The basic oxide of Al_2O_3, MgO and CaO were selected as the electrolyte additive of electrolytic Zn-Mn batteries. As shown in **Fig. S22A, B**, although these different electrolytes all exhibited stable gel-like feature, the brown precipitate was observed in the MgO and CaO electrolyte. Obviously, the $Mn(OH)_2$ or other manganese oxides were deposited in the MgO and CaO electrolyte, which may attributed to the excessively high pH levels of electrolyte (K^{θ}_{sp} of $Mn(OH)_2$ is 4×10^{-14}, the deposition pH is around 8). The insoluble substances in different electrolyte were collected, and the corresponding XRD patterns were shown in the **Fig. S22C**. Without obvious characteristic diffraction peaks of ZSH can be indexed in theses electrolyte. For the CaO gel-like electrolyte, the $CaSO_4 \cdot 2H_2O$ was deposited. Subsequently, the charge-discharge capacities of these different gel-like electrolyte was recorded and shown in the **Fig. S22D**. The batteries with CaO and MgO gel-like electrolyte exhibit negligible charge capacity after 30 min chronoamperometric charge under 2.0 V *vs.* Zn/Zn^{2+}, which may attributed to the deposition of $Mn(OH)_2$ and Other impurities in the electrolyte. For the Al_2O_3 gel-like electrolyte, the charge capacities are lower than the 1 M $ZnSO_4$ +2 M $MnSO_4$ electrolyte during the initial 20 cycles.

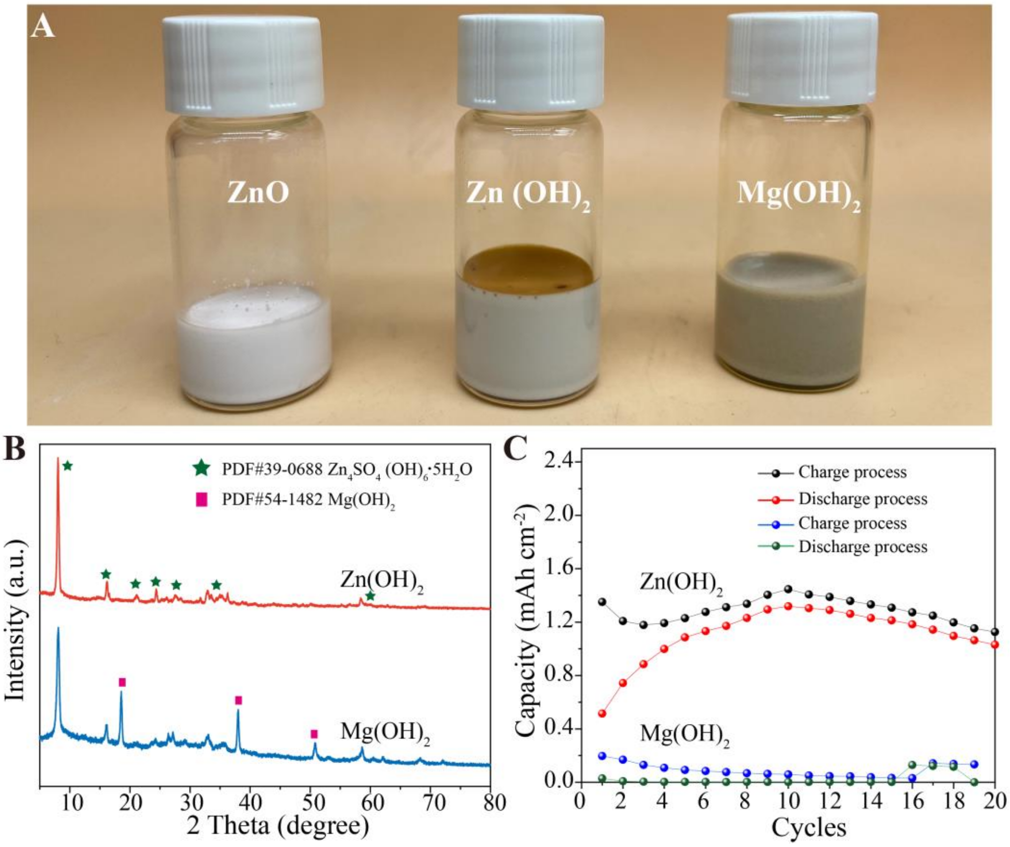

Fig. S23 (**A**) the digital image of papered ZnO-based, $Zn(OH)_2$-based and $Mg(OH)_2$-based electrolyte. (**B**) The XRD patterns of insoluble substance in the $Zn(OH)_2$ and $Mg(OH)_2$. (**C**) Charge and discharge capacities of different button cell electrolyte Zn-Mn batteries during the initial 20 cycles, the chronoamperometric charge of 2.0 V *vs.* Zn/Zn^{2+} is 30 min, and the discharge current is 1 mA cm^{-2}

We further prepared the $Zn(OH)_2$ and $Mg(OH)_2$ based gel-like electrolyte by using $Zn(OH)_2$ and $Mg(OH)_2$ as the additive in the 1 M $ZnSO_4$ + 2 M $MnSO_4$ electrolyte, and the concentration is 0.2g mL^{-1}. As shown in the **Fig. S23A**, the Color of $Zn(OH)_2$ and $Mg(OH)_2$ gel-like electrolyte is brown, which attributed to the deposition of $Mn(OH)_2$ or other manganese oxides. the XRD pattern of insoluble substance of the $Zn(OH)_2$ and $Mg(OH)_2$ electrolyte were collected and shown in **Fig. S23B**. The characteristic diffraction peak of $Zn_4SO_4(OH)_6{\cdot}5H_2O$ (ZSH; PDF # 39-0688) can be detected in the $Zn(OH)_2$ and $Mg(OH)_2$ electrolyte. At the same time, the $Mg(OH)_2$ phase also can be detected in the $Mg(OH)_2$ electrolyte.

The electrochemical performance of $Zn(OH)_2$ and $Mg(OH)_2$ electrolyte were tested using the CR2032 button cell mold. During the initial 10 cycles, the charge capacity of $Zn(OH)_2$ electrolyte is continually increased, and can reach 1.44 mAh cm^{-2} (it is higher than conventional aqueous electrolyte, but much lower than ZnO gel-like electrolyte). For the $Mg(OH)_2$ electrolyte, after 30 min chronoamperometric charge under 2.0 V vs. Zn/Zn^{2+}, the charge capacity is negligible, which may attributed to the presence of insoluble $Mg(OH)_2$ in the electrolyte.

According to the **Figs. S22-S23**, when selecting electrolyte additives, it is crucial to carefully evaluate their effect on the pH range of the electrolyte. Significant fluctuations in pH could lead to the formation of $Mn(OH)_2$ or other manganese oxides precipitate, which can adversely impact the electrochemical performance of the batteries. Furthermore, the insoluble impurities in the electrolyte also can limit the electrochemical performance of batteries.

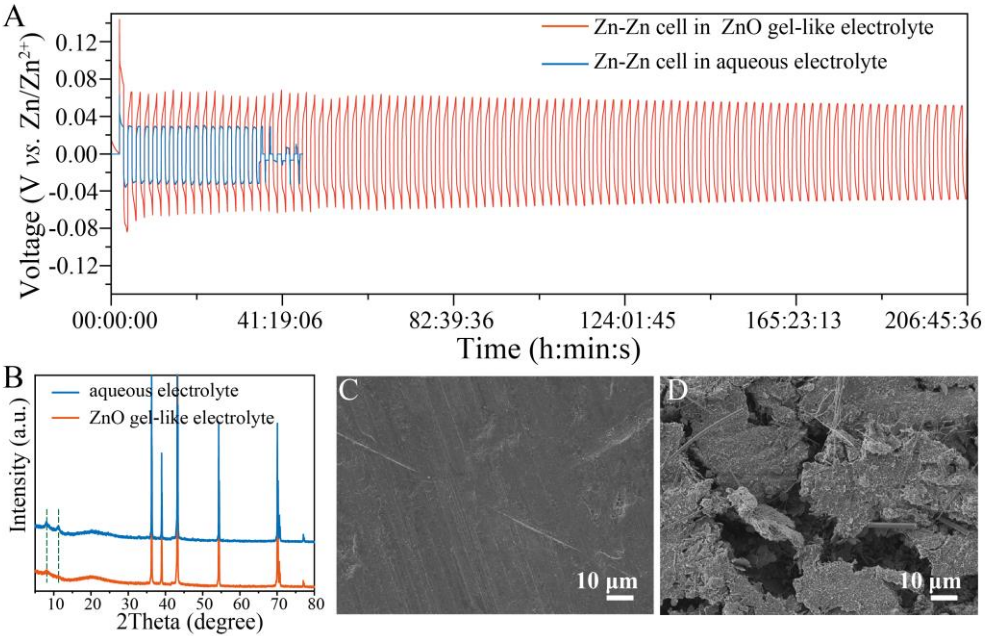

Fig. S24 (**A**) Comparison of the cycling stability of Zn-Zn symmetric cell in ZnO gel-like electrolyte and 1 M $ZnSO_4$ + 2M $MnSO_4$ electrolyte (**B**) the XRD patterns of the Zn metal after 10 plating/stripping process. SEM images of the Zn metal after 10 after 10 plating/stripping process in (**C**)ZnO gel-like electrolyte and (**D**) conventional aqueous electrolyte. the current density is 1 mA cm^{-2}, and the capacity is 1 mA h cm^{-2}

To investigate the effect of ZnO electrolyte addition on the growth of Zn dendrites of Zn metal anode, the Zn//Zn symmetric cells were assembled using ZnO gel-like electrolyte and a CR2032 coin-type cell mold. As shown in **Fig. S24A**, in ZnO gel-like electrolyte, the plating/stripping performance of Zn//Zn symmetric cell exhibits a stable polarization voltage profile over 200h without obvious fluctuation at a current density of 1mA cm^{-2} with an area capacity of 1mAh cm^{-2}. In contrast, when using 1 M $ZnSO_4$ +2 M $MnSO_4$ solution as the electrolyte, the Zn//Zn cell exhibits a sudden reduction of the polarization voltage after cycling for 38 h at 1mA cm^{-2}, which ascribe to a dynamic dendrite-induced short circuit. After 10 plating/stripping process of 1mA cm^{-2} with 1mAh cm^{-2}, the surface of the metal zinc was observed by SEM. As shown in **Fig. S24D,** in conventional 1 M $ZnSO_4$ +2 M $MnSO_4$ electrolyte, the cliffy dendrite pieces and dark by-product aggregation on Zn foil were caught. Once switched to ZnO gel-like electrolyte., the surface of Zn metal became much smoother than the previous one (**Fig. S24C**).

Based on the experimental results presented above, it can be unequivocally concluded that the ZnO gel-like electrolyte can suppress the growth of Zn dendrites. Through extensive research in the previous published works, we found that the ZSH is extensively utilized by many researchers as a protective coating for the metal Zn anode, Thus, according to the published references and our in-situ differential electrochemical mass spectrometry (DEMS) gas analysis (**Fig. 2E**), **Figures S6** and **S7**, the improved cycling ability of Zn metal anode in ZnO gel-like electrolyte may mainly attributed to the following points;

1) the ZnO gel-like electrolyte can alleviate corrosion of zinc metal (decreased HER reactivity).
2) The ZSH on the anode side can form a protective layer on the surface of Zn metal anode.
3) The ZSH layer shows high electrochemical stability, mechanical robustness, and active edges, which can effectively suppress the electrolyte-introduced side reactions and dendrite growth [S5-S7].

Table S4 Summary of the parameters and electrochemical performance of aqueous electrolytic Zn-Mn batteries.

Electrolyte	Cathode	Max Charge capacity	Charge rate	Retention/Cycles (Charge Capacity/ discharge rate)	References
Acidic electrolytes with Mn^{2+}/MnO_2 reactions					
1M $MnSO_4$ + 0.5 M H_2SO_4	Carbon cloth	0.4 mAh cm^{-2}	---	97.5%/6000 cycles (0.4 mAh cm^{-2}/2.5mA cm^{-2})	[S8]
1M $MnSO_4$ + 1M $ZnSO_4$ + 1 M H_2SO_4 +PVA	Carbon felt	3.3 mAh cm^{-2}	---	92%/200 cycles (0.3 mAh cm^{-2}/1.3 mA cm^{-2})	[S9]
3 M $MnSO_4$ + 0.3 M H_2SO_4 + 0.06 $NiSO_4$	Carbon felt	1 mAh cm^{-2}	---	96.6%/600 cycles (1 mAh cm^{-2}/10 mA cm^{-2})	[S10]
1M $MnSO_4$ + 1M $ZnSO_4$ + 0.1 M H_2SO_4 + 0.07 mM PVP	Carbon felt	4 mAh cm^{-2}	---	90%/2000 cycles (50 Wh m^{-2}/20 C)	[S11]
1M $MnSO_4$ + 1M $ZnSO_4$ + 0.3 M H_2SO_4	Carbon cloth	2 mAh cm^{-2}	---	92%/1600 cycles (2 mAh cm^{-2}/30 mA cm^{-2})	[S12]
1M $MnSO_4$ + 1M $ZnSO_4$ + 0.2 M HAc	Carbon cloth	3 mAh cm^{-2}	---	99%/2000 cycles (0.8 mAh cm^{-2}/10 mA cm^{-2}	[S13]

1M $ZnSO_4$ + 1M $MnCl_2$ + 0.5 M H_2SO_4	Carbon cloth	0.5 mAh cm^{-2} (90% CE (2.2 V *vs.* Zn/Zn^{2+}))	---	95%/1600 cycles (0.5 mAh cm^{-2}/5mA cm^{-2})	[S14]
Mild aqueous electrolyte					
2M $ZnSO_4$ + 0.2M $MnSO_4$	Ca-PTA·$2H_2O$	0.51 mAh cm^{-2}	0.06 mA cm^{-2}	70%/70 cycles (0.06 mA cm^{-2})	[S15]
2M $ZnSO_4$ + 0.5M $MnSO_4$	Acidic CNTs	0.9 mAh cm^{-2}	0.1 mA cm^{-2}	---	[S16]
2 M $ZnSO_4$ + 0.5 M $MnSO_4$ + 0.25 M $Al_2(SO_4)_3$	graphite foil	2 mAh cm^{-2}	5 mA cm^{-2}	---	[S17]
2M $ZnSO_4$ + 0.2M $MnSO_4$	MOFs-73	0.84 mAh cm^{-2}	0.05 mA cm^{-2}	73%/ 50 cycles (0.05 mA cm^{-2})	[S18]
2M $ZnSO_4$ + 0.2M $MnSO_4$	Bi-PYDC	0.25 mAh cm^{-2}	0.1 mA cm^{-2}	48%/800 cycles (0.1 mA cm^{-2})	[S19]
3M $ZnSO_4$ + 0.3M $MnSO_4$	Co_3O_4	0.6 mAh cm^{-2}	0.5 mA cm^{-2}	---	[S20]
1M $ZnSO_4$ + 1M LiCl+ 5 mM $MnSO_4$	Carbon cloth	0.4 mAh cm^{-2}	2.5 mA cm^{-2}	---	[S21]

The symbol of --- represent that no related information was found in the reference.

Table S5 Summary comparison of electrochemical performance of Zn-based energy storage systems based on the mass of active material in cathode

Cathode material	Electrolyte	Average charge current (mA cm^{-2})	Active substance load (mg cm^{-2})	Specific capacity (mAh cm^{-2})	Ref.
Carbon nanotubes film	ZnO gel-like electrolyte	5.46 (max)	---	2.5	This work
γ-MnO_2	$Zn(CF_3SO_3)_2$ in PC+EC+PVDF	0.01	3	0.315	[S22]
Birnessite MnO_2	0.25 M $ZnSO_4$ +0.75 M Na2SO4	0.77	2.5	0.7625	[S23]
CC/NCNTs/δ-MnO_2	2 M $ZnSO_4$ +0.2 M $MnSO_4$	0.4	4	1.95	[S24]

C–MnO_2	PAM-PEI	1	5	1.3	[S25]
CT/SWNT		1.2	12	1.37	[S26]
MCV@CC	3M $Zn(CF_3SO_3)_2$	1	7	2.36	[S27]
Mn_3O_4	1 M $ZnSO_4$ + 1 M $MnSO_4$	0.2	2	0.441	[S28]
Mn_3O_4@C	1 M $ZnSO_4$ + 0.5 M $NaSO_4$ + 0.1 M $MnSO_4$	2.5	2.5	0.8	[S29]
MnO_2 /CNT	2 M $ZnSO_4$ + 0.2 M $MnSO_4$ saturated Cellulose/Polyacryl amide	0.924	3	0.78	[S30]
MnO_2 /CNT	3 M $ZnSO_4$+ 0.1 M $MnSO_4$+ 20 wt% xanthan gum	0.15	0.5	0.13	[S31]
MnO_2/CNT	GE	0.154	2.5	0.765	[S32]
MnO_2 @PEDOT	PVA/ZnCl 2 /MnSO 4	3.7	5	1.97	[S33]
MnO_2@PEDOT	3 M LiCl+ 2 M $ZnCl_2$ + 0.4 M $MnSO_4$	4	1.78	1.97	[S34]
MnO_2	2 M $ZnSO_4$ + 0.2 M $MnSO_4$	0.75	2.5	0.722	[S35]
MnO_2/rGO	2 M $ZnSO_4$ + 0.1 M $MnSO_4$	0.9	3	0.996	[S36]
N-CC@MnO_2	3 M LiCl + 2 M $ZnCl_2$ + 0.4 M $MnSO_4$	1.6	3.2	1.13	[S37]
PANI-MnO_2	2 M $ZnSO_4$ + 0.1 M $MnSO_4$	0.1	2	0.596	[S38]
Spinel $ZnMn_2O_4$@C	3M $Zn(CF_3SO_3)_2$	0.09	1.8	0.27	[S39]
todorokiteMnO_2	1 M $ZnSO_4$	0.1	2	0.208	[S40]
$ZnMn_2O_4$@P EDOT	1 M $ZnSO_4$	0.5	6.2	1.2834	[S41]
ZSH	2 M $ZnSO_4$ + 0.24 M $MnSO_4$	0.25	5	0.77	[S42]
α-MnO_2	1 M $ZnSO_4$	0.0512	3.2	1.1296	[S43]
α-MnO_2	1 M $ZnSO_4$	0.0525	5	1.025	[S44]
α-MnO_2	1 M $ZnSO_4$	0.0525	5	0.975	[S45]
α-MnO_2 on 3DC	2 M $ZnCl_2$ +0.4 M $MnSO_4$	0.16	3.2	1.1296	[S46]
β-MnO_2	1 M $ZnSO_4$ +0.1 M $MnSO_4$	0.168	1.68	0.4536	[S47]
δ-MnO_2	0.5M $Zn(CF_3SO_3)_2$ in acetonitrile	0.0246	2	0.24	[S48]
λ-MnO_2	1 M $ZnSO_4$	0.0952	7	3.0982	[S49]
$Ca_{0.25}V_2O_5$	1 M $ZnSO_4$	0.3876	5.7	1.938	[S50]
$Cu_{0.18}V_2O_5\cdot 0.72H_2O$@CC	3 M $Zn(CF_3SO_3)_2$	0.7	7	1.63	[S51]
$H_2V_3O_8$	3 M $Zn(CF_3SO_3)_2$	0.12	1.2	0.50856	[S52]

$K_2V_6O_{16}$•2.7H $_2$O	1 M $ZnSO_4$	0.3	3	0.717	[S53]
$Mg_{0.34}V_2O_5$•0. 84H_2O	3 M $Zn(CF_3SO_3)_2$	0.35	7	2.471	[S54]
$Na_{0.33}V_2O_5$	3 M $Zn(CF_3SO_3)_2$	0.4	2	0.746	[S55]
$Na_{1.1}V_3O_{7.9}$@r GO	1 M $Zn(CF_3SO_3)_2$	0.36	1.2	0.264	[S56]
$Na_2V_6O_{16}$•3H_2O	1 M $ZnSO_4$	0.35	3.5	1.2635	[S57]
$Na_3V_2(PO_4)_3F_3$@C	2 M $Zn(CF_3SO_3)_2$	0.8	10	0.75	[S58]
TiN @ V_2O_5NWA / CNTF	$ZnSO_4$-PVA	0.5	0.52	1.45	[S59]
$V_{10}O_{24}$•12H_2O	3 M $Zn(CF_3SO_3)_2$	0.1	2	0.329	[S60]
V_2O_5	3 M $Zn(CF_3SO_3)_2$	1	5	2.28	[S61]
V_2O_5•nH_2O	0.5M $Zn(CF_3SO_3)_2$ in acetonitrile	0.04608	3.2	0.6272	[S62]
VO_2/rGO	3 M $Zn(CF_3SO_3)_2$	0.15	1.5	0.414	[S63]
$Zn_2(OH)VO_4$	4% fumed silica into 2M ZnSO4	0.41	4.1	0.8364	[S64]
$Zn_2V_2O_7$	1 M $ZnSO_4$	0.175	3.5	0.868	[S65]
$Zn_3V_2O_7(OH)_2$•$_2H_2O$	1 M $ZnSO_4$	0.25	5	1.0605	[S66]
FeHCF	1M $Zn(CH_3COO)_2$ + choline acetate with 30 wt % of water	0.1	20	1.08	[S67]
FeHCF	1M $Zn(CH_3COO)_2$ + choline acetate with 30 wt % of water	0.05	5	0.61	[S68]
NiHCF	0.5 M $Zn(ClO_4)_2$ In acetonitrile	0.02486	2.22	0.1234	[S69]
ZnHCF	1M $ZnSO_4$	0.48	8	0.5232	[S70]
ZnHCF	3M $ZnSO_4$	0.48	8	0.532	[S71]
ZnHCF@MnO_2	0.5M $ZnSO_4$	0.12	1.2	0.1416	[S72]
FNCH	3 M KOH	1	2.14	0.23	73]
ZCNS/NF	5M KOH + 0.1M $Zn(CF_3SO_3)_2$	12	4	2.3	[S74]
$Co_xNi_{3-x}S_2$	3 M KOH+0.15 M $Zn(Ac)_2$	5	5	2.45	[S75]
CNF@$NiCo_2S_4$	3 MKOH + 0.1M $Zn(CH_3COO)_2$	2	1.58	0.32	[S76]
NF\Ni_3S_2/NiS@NiCo-LDH	6 M KOH	3	4.2	1.73	[S77]

Supplementary References

[S1] Ivette Aguilar, Pierre Lemaire, Nawfel Ayouni, Ezzoubair Bendadesse, Anatolii V Morozov, Ozlem Sel, Véronique Balland, Benoît Limoges, Artem M Abakumov, Encarnacion Raymundo-Piñero. Energy Storage Mater. 53, 238 (2022). https://doi.org/10.1016/j.energy.2021.121746

[S2] Denise Cardoso, Agnès Narcy, Stéphane Durosoy, Yves Chevalier, Colloid. Surface. A 650, 129653 (2022). https://doi.org/10.1016/j.colsurfa.2022.129653

[S3] Mohd Omar Fatehah, Hamidi Abdul Aziz, Serge Stoll, J. Colloid Sci. Biotechnol., 3, 75, (2014) https://doi.org/10.1166/jcsb.2014.1072

[S4] X. Guo, H.-S. Xiao, F. Wang, Y.-H. Zhang, The J. Phys. Chem. A, 114 (2010) 6480-6486. https://doi.org/10.1021/jp9104147

[S5] Y. Liu, S. Chen, H. Yuan, F. Xiong, Q. Liu, Y. An, J. Zhang, L. Wu, J. Sun, Y-W Zhang, Sci. Bull., (2023). https://doi.org/10.1016/j.scib.2023.09.034

[S6] M. Peng, Z. Zhu, K. Yang, L. Lv, Y. Duan, X. Liao, X. He, G. You, R. Song, L. He. Chem. Eng. J., 468, 143561 (2023) https://doi.org/10.1016/j.cej.2023.143561

[S7] W. Yuan, G. Ma, X. Nie, Y. Wang, S. Di, L. Wang, J. Wang, S. Shen, N. Zhang, Chem. Eng. J., 431, 134076 (2022) https://doi.org/10.1016/j.cej.2021.134076

[S8] C. Liu, X. Chi, Q. Han, Y. Liu, Adv. Energy Mater, 10, 1903589 (2020) https://doi.org/10.1002/aenm.201903589

[S9] Y. Yuan, J. Yang, Z. Liu, R. Tan, M. Chuai, J. Sun, Y. Xu, X. Zheng, M. Wang, T. Ahmad, Adv. Energy Mater., 12, 2103705 (2022) https://doi.org/10.1002/aenm.202103705

[S10] D. Chao, C. Ye, F. Xie, W. Zhou, Q. Zhang, Q. Gu, K. Davey, L. Gu, S.Z. Qiao, Adv. Mater., 32 ,2001894 (2020) https://doi.org/10.1002/adma.202001894

[S11] M. Chuai, J. Yang, R. Tan, Z. Liu, Y. Yuan, Y. Xu, J. Sun, M. Wang, X. Zheng, N. Chen, Adv. Mater., 34, 2203249 (2022) https://doi.org/10.1002/adma.202203249

[S12] D. Chao, W. Zhou, C. Ye, Q. Zhang, Y. Chen, L. Gu, K. Davey, S.Z. Qiao, Angew Chem Int Edit, 58, 7823 (2019) https://doi.org/10.1002/anie.201904174

[S13] Z. Liu, Y. Yang, S. Liang, B. Lu, J. Zhou. Small Struct., 2, 2100119 (2021) https://doi.org/10.1002/sstr.202100119

[S14] L. Yang, L. Meng, X. Ji, S. Cheng. Batteries & Supercaps, 6, e202300158 (2023) https://doi.org/10.1002/batt.202300158

[S15] W. Gou, T. Jiang, W. Wang, Q. Fan, Y. Zhang. Chinese Chem. Letters, 34, 107760 (2023) https://doi.org/10.1016/j.cclet.2022.107760

[S16] H. Chen, H. Kuang, F. Liu, Y. Wu, S. Cai, M. Xu, S.-J. Bao. J. Colloid Inter. Sci., 600, 83-89 (2021) https://doi.org/10.1016/j.jcis.2021.04.097

[S17] Z. Qin, Y. Song, D. Yang, M.-Y. Zhang, H.-Y. Shi, C. Li, X. Sun, X.-X. Liu. ACS Appl. Mate. Interfaces, **14**, 10526-10534 (2022) https://doi.org/10.1021/acsami.1c22674

[S18] W. Gou, H. Chen, Z. Xu, Y. Sun, X. Han, M. Liu, Y. Zhang. Energy Adv., **1**, 1065-1070 (2022) https://doi.org/10.1039/D2YA00257D

[S19] L. Gou, J. Li, K. Liang, S. Zhao, D. Li, X. Fan. Small, **19**, 2208233 (2023) https://doi.org/10.1002/smll.202208233

[S20] Z. Li, Y. Li, X. Ren, Y. Zhao, Z. Ren, Z. Yao, W. Zhang, H. Xu, Z. Wang, N. Zhang. Small, 2301770 (2023) https://doi.org/10.1002/smll.202301770

[S21] N. Chen, W. Wang, Y. Ma, M. Chuai, X. Zheng, M. Wang, Y. Xu, Y. Yuan, J. Sun, K. Li. Small Methods, 2201553 (2023) https://doi.org/10.1002/smtd.202201553

[S22] X. Guo, H.-S. Xiao, F. Wang, Y.-H. Zhang, The J. Phys. Chem. A, 114 (2010) 6480-6486. https://doi.org/10.1021/jp9104147

[S23] G.G. Kumar, S. Sampath, Solid State Ionics, 160 (2003) 289-300. https://doi.org/10.1016/S0167-2738(03)00209-1

[S24] J.S. Ko, M.B. Sassin, J.F. Parker, D.R. Rolison, Jeffrey W. Long, Sustainable Energy Fuels, 2 (2018) 626-636. https://doi.org/10.1039/C7SE00540G

[S25] M. Zhao, Y. Luo, L. Zhu, D. Cai, Y. Zhuang, Q. Chen, H. Zhan,J. Alloys Compd., 913 (2022) 165124. https://doi.org/10.1016/j.jallcom.2022.165124

[S26] F. Li, Y.-L. Liu, G.-G. Wang, D. Yan, G.-Z. Li, H.-X. Zhao, H.-Y. Zhang, H.-Y. Yang, J. Mater. Chem. A, 9 (2021) 9675-9684. https://doi.org/10.1039/D0TA12009J

[S27] Y. Cao, X. Tang, M. Liu, Y. Zhang, T. Yang, Z. Yang, Y. Yu, Y. Li, J. Di, Q. Li, Chem. Eng. J., 431 (2022) 133241. https://doi.org/10.1016/j.cej.2021.133241

[S28] K. Fang, F. Li, G.-G. Wang, Y.-L. Liu, M.-L. Tan, D.-Q. Zhao, H.-Y. Zhang, J.-C. Han,J. Mater. Sci. Technol., 143 (2023) 84-92. https://doi.org/10.1016/j.jmst.2022.09.042

[S29] L. Wang, X. Cao, L. Xu, J. Chen, J. Zheng, ACS Sustainable Chem. Eng., 6 (2018) 16055-16063. https://doi.org/10.1021/acssuschemeng.8b02502

[S30] Q. Zhao, W. Huang, Z. Luo, L. Liu, Y. Lu, Y. Li, L. Li, J. Hu, H. Ma, J. Chen, Sci. Adv., 4 (2018) eaao1761. https://doi.org/10.1126/sciadv.aao1761

[S31] D. Wang, H. Li, Z. Liu, Z. Tang, G. Liang, F. Mo, Q. Yang, L. Ma, C. Zhi, Small, 14 (2018) 1803978. https://doi.org/10.1002/smll.201803978

[S32] S. Zhang, N. Yu, S. Zeng, S. Zhou, M. Chen, J. Di, Q. Li, J. Mater. Chem. A, 6 (2018) 12237-12243. https://doi.org/10.1039/C8TA04298E

[S33] H. Li, C. Han, Y. Huang, Y. Huang, M. Zhu, Z. Pei, Q. Xue, Z. Wang, Z. Liu, Z. Tang, Y. Wang, F. Kang, B. Li, C. Zhi,Energy Environ. Sci., 11 (2018) 941-951. https://doi.org/10.1039/C7EE03232C

[S34] Y. Zeng, X. Zhang, Y. Meng, M. Yu, J. Yi, Y. Wu, X. Lu, Y. Tong, Adv. Mater., 29 (2017) 1700274. https://doi.org/10.1002/adma.201700274

[S35] S. Park, G.H. An, Int. J. Energy Res., 46 (2022) 8464-8470. https://doi.org/10.1002/er.7687

[S36] Y. Huang, J. Liu, Q. Huang, Z. Zheng, P. Hiralal, F. Zheng, D. Ozgit, S. Su, S. Chen, P.-H. Tan, S. Zhang, H. Zhou, npj Flexible Electron., 2 (2018) 21. https://doi.org/10.1038/s41528-018-0034-0

[S37] W. Qiu, Y. Li, A. You, Z. Zhang, G. Li, X. Lu, Y. Tong, J. Mater. Chem. A, 5 (2017) 14838-14846. https://doi.org/10.1039/C7TA03274A

[S38] J. Huang, Z. Wang, M. Hou, X. Dong, Y. Liu, Y. Wang, Y. Xia, Nat. Commun., 9 (2018) 2906. https://doi.org/10.1038/s41467-018-04949-4

[S39] N. Zhang, F. Cheng, Y. Liu, Q. Zhao, K. Lei, C. Chen, X. Liu, J. Chen, J. Am. Chem. Soc., 138 (2016) 12894-12901. https://doi.org/10.1021/jacs.6b05958

[S40] J. Lee, J.B. Ju, W.I. Cho, B.W. Cho, S.H. Oh, Electrochim. Acta, 112 (2013) 138-143. https://doi.org/10.1016/j.electacta.2013.08.136

[S41] H. Zhang, J. Wang, Q. Liu, W. He, Z. Lai, X. Zhang, M. Yu, Y. Tong, X. Lu, Energy Storage Mater., 21 (2019) 154-161. https://doi.org/10.1016/j.ensm.2018.12.019

[S42] S. Zhao, B. Han, D. Zhang, Q. Huang, L. Xiao, L. Chen, D.G. Ivey, Y. Deng, W. Wei, J. Mater. Chem. A, 6 (2018) 5733-5739. https://doi.org/10.1039/C8TA01031E

[S43] M.H. Alfaruqi, J. Gim, S. Kim, J. Song, J. Jo, S. Kim, V. Mathew, J. Kim, J. Power Sources, 288 (2015) 320-327. https://doi.org/10.1016/j.jpowsour.2015.04.140

[S44] B. Lee, C.S. Yoon, H.R. Lee, K.Y. Chung, B.W. Cho, S.H. Oh, Sci. Rep., 4 (2014) 6066. https://doi.org/10.1038/srep06066

[S45] B. Lee, H.R. Lee, H. Kim, K.Y. Chung, B.W. Cho, S.H. Oh, Chem. Commun., 51 (2015) 9265-9268. https://doi.org/10.1039/C5CC02585K

[S46] W. Qiu, Y. Li, A. You, Z. Zhang, G. Li, X. Lu, Y. Tong, Journal of Materials Chemistry A, 5 (2017) 14838-14846. https://doi.org/10.1039/C7TA03274A

[S47] S. Islam, M.H. Alfaruqi, V. Mathew, J. Song, S. Kim, S. Kim, J. Jo, J.P. Baboo, D.T. Pham, D.Y. Putro, Y.-K. Sun, J. Kim, J. Mater. Chem. A, 5 (2017) 23299-23309. https://doi.org/10.1039/C7TA07170A

[S48] S.-D. Han, S. Kim, D. Li, V. Petkov, H.D. Yoo, P.J. Phillips, H. Wang, J.J. Kim, K.L. More, B. Key, R.F. Klie, J. Cabana, V.R. Stamenkovic, T.T. Fister, N.M. Markovic, A.K. Burrell, S. Tepavcevic, J.T. Vaughey, Chem. Mater., 29 (2017) 4874-4884. https://doi.org/10.1021/acs.chemmater.7b00852

[S49] C. Yuan, Y. Zhang, Y. Pan, X. Liu, G. Wang, D. Cao, Electrochim. Acta, 116 (2014) 404-412. https://doi.org/10.1016/j.electacta.2013.11.090

[S50] C. Xia, J. Guo, P. Li, X. Zhang, H.N. Alshareef, Angew. Chem. Int. Ed., 57 (2018) 3943-3948. https://doi.org/10.1002/ange.201713291

[S51] J. Ren, P. Hong, Y. Ran, Y. Chen, X. Xiao, Y. Wang, Inorg. Chem. Front., 9 (2022) 792-804. https://doi.org/10.1039/D1QI01499D

[S52] P. He, Y. Quan, X. Xu, M. Yan, W. Yang, Q. An, L. He, L. Mai, Small, 13 (2017) 1702551. http://doi.org/10.1002/sl.201702551

[S53] B. Sambandam, V. Soundharrajan, S. Kim, M.H. Alfaruqi, J. Jo, S. Kim, V. Mathew, Y.-k. Sun, J. Kim, J. Mater. Chem. A, 6 (2018) 15530-15539. https://doi.org/10.1039/C8TA02018C

[S54] F. Ming, H. Liang, Y. Lei, S. Kandambeth, M. Eddaoudi, H.N. Alshareef, ACS Energy Lett., 3 (2018) 2602-2609. https://doi.org/10.1021/acsenergylett.1c01512

[S55] P. He, G. Zhang, X. Liao, M. Yan, X. Xu, Q. An, J. Liu, L. Mai, Adv. Energy Mater., 8 (2018) 1702463. https://doi.org/10.1002/aenm.201702463

[S56] Y. Cai, F. Liu, Z. Luo, G. Fang, J. Zhou, A. Pan, S. Liang, Energy Storage Mater., 13 (2018) 168-174. https://doi.org/10.1016/j.ensm.2018.01.009

[S57] V. Soundharrajan, B. Sambandam, S. Kim, M.H. Alfaruqi, D.Y. Putro, J. Jo, S. Kim, V. Mathew, Y.-K. Sun, J. Kim, Nano Lett., 18 (2018) 2402-2410. https://doi.org/10.1021/acs.nanolett.7b05403

[S58] W. Li, K. Wang, S. Cheng, K. Jiang, Energy Storage Mater., 15 (2018) 14-21. https://doi.org/10.1016/j.ensm.2018.03.003

[S59] Q. Li, Q. Zhang, C. Liu, Z. Zhou, C. Li, B. He, P. Man, X. Wang, Y. Yao, J. Mater. Chem. A, 7 (2019) 12997-13006. https://doi.org/10.1039/C9TA03330K

[S60] T. Wei, Q. Li, G. Yang, C. Wang, Electrochim. Acta, 287 (2018) 60-67. https://doi.org/10.1016/j.electacta.2018.08.040

[S61] N. Zhang, Y. Dong, M. Jia, X. Bian, Y. Wang, M. Qiu, J. Xu, Y. Liu, L. Jiao, F. Cheng, ACS Energy Lett., 3 (2018) 1366-1372. https://doi.org/10.1021/acsenergylett.8b00565

[S62] P. Senguttuvan, S.-D. Han, S. Kim, A.L. Lipson, S. Tepavcevic, T.T. Fister, I.D. Bloom, A.K. Burrell, C.S. Johnson, Adv. Energy Mater., 6 (2016) 1600826. https://doi.org/10.1002/aenm.201600826

[S63] X. Dai, F. Wan, L. Zhang, H. Cao, Z. Niu, Energy Storage Mater., 17 (2019) 143-150. https://doi.org/10.1016/j.ensm.2018.07.022

[S64] D. Chao, C. Zhu, M. Song, P. Liang, X. Zhang, N.H. Tiep, H. Zhao, J. Wang, R. Wang, H. Zhang, H.J. Fan, Adv. Mater., 30 (2018) 1803181. https://doi.org/10.1002/adma.201803181

[S65] B. Sambandam, V. Soundharrajan, S. Kim, M.H. Alfaruqi, J. Jo, S. Kim, V.

Mathew, Y.-k. Sun, J. Kim, J. Mater. Chem. A, 6 (2018) 3850-3856. https://doi.org/10.1039/C7TA11237H

[S66] C. Xia, J. Guo, Y. Lei, H. Liang, C. Zhao, H.N. Alshareef, Adv. Mater., 30 (2018) 1705580. https://doi.org/10.1002/adma.201705580

[S67] Z. Liu, P. Bertram, F. Endres, J. Solid State Electrochem., 21 (2017) 2021-2027. https://doi.org/10.1007/s10008-017-3589-0

[S68] Z. Liu, G. Pulletikurthi, F. Endres, ACS Appl. Mater. Interfaces, 8 (2016) 12158-12164. https://doi.org/10.1021/acsami.6b01592

[S69] M.S. Chae, J.W. Heo, H.H. Kwak, H. Lee, S.-T. Hong, J. Power Sources, 337 (2017) 204-211. https://doi.org/10.1016/j.jpowsour.2016.10.083

[S70] L. Zhang, L. Chen, X. Zhou, Z. Liu, Adv. Energy Mater., 5 (2015) 1400930. https://doi.org/10.1002/aenm.201400930

[S71] L. Zhang, L. Chen, X. Zhou, Z. Liu, Sci. Rep., 5 (2015) 18263. https://doi.org/10.1038/srep18263

[S72] K. Lu, B. Song, Y. Zhang, H. Ma, J. Zhang, J. Mater. Chem. A, 5 (2017) 23628-23633. https://doi.org/10.1039/C7TA07834J

[S73] W. Liu, Q. Zhao, Y. Wang, Y. Chen, L. Chen, Nanomaterials, 12 (2022) 1780. https://doi.org/10.3390/nano12101780

[S74] Y. Zhou, X. Tong, N. Pang, Y. Deng, C. Yan, D. Wu, S. Xu, D. Xiong, L. Wang, P.K. Chu, ACS Appl. Mater. Interfaces, 13 (2021) 34292-34300. https://doi.org/10.1021/acsami.1c08108

[S75] W. He, S. Wang, Y. Shao, Z. Kong, H. Tu, Y. Wu, X. Hao, Adv. Energy Mater., 11 (2021) 2003268. https://doi.org/10.1002/aenm.202003268

[S76] Z. Cui, S. Shen, J. Yu, J. Si, D. Cai, Q. Wang, Chem. Eng. J., 426 (2021) 130068. https://doi.org/10.1016/j.cej.2021.130068

[S77] K. Zhou, S. Wang, G. Zhong, J. Chen, Y. Bao, L. Niu, Small, 18 (2022) 2202799. https://doi.org/10.1002/smll.202202799
